# Supplementary material for: A Module-Level Polygenic Risk Score-Based NetWAS Framework for Identifying AD Genetic Modules Mediated by Amygdala: An ADNI Study
Source: Int J Mol Sci. 2025 Jun 24;26(13):6060. doi: 10.3390/ijms26136060 (PMC12249635; doi:10.3390/ijms26136060)
Supplement: Supplementary file 1 [file ijms-26-06060-s001.zip › ijms-3668290-supplementary.pdf]

Supplementary Materials for "A module-level polygenic risk score-based NetWAS framework for identifying AD genetic modules mediated by amygdala: an ADNI study" by Haoran Luo, Shaoheng Fan, Hongwei Liu, Wei Li, Zhoujie Fan, Xuancheng Zhu, Chen Jason Zhang, Hong Liang, Shan Cong, Xiaohui Yao.

S1 Supplementary Materials and Methods

S1.1 Visualization and interpretation of amygdala-related mechanisms in AD

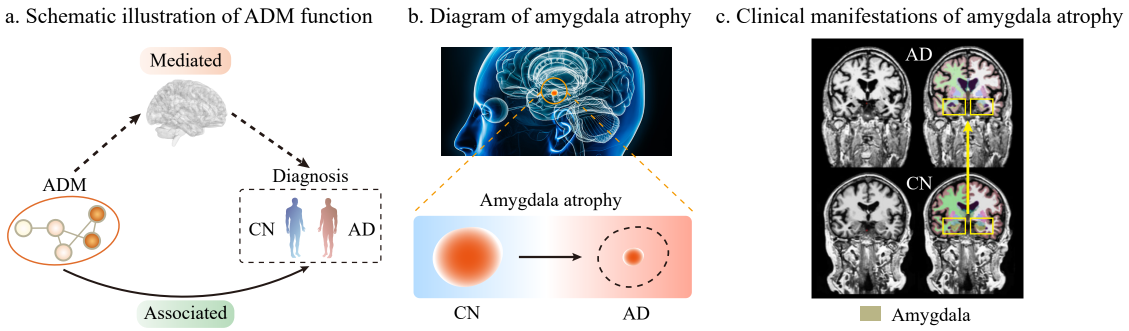

Figure S1: Visualization of ADM function and clinical phenotype. (**a**) Schematic illustration of the functional role of AD-relevant modules (ADMs) in the imaging genetics framework. ADMs are associated with clinical diagnosis (CN vs. AD) and mediated through amygdala-based intermediate quantitative traits (iQTs). (**b**) Diagram of amygdala atrophy, showing a reduction in amygdala volume from CN to AD status. (**c**) Clinical brain imaging comparison between AD and CN individuals, highlighting visible atrophy of the amygdala region (highlighted in green) in coronal structural MRI scans. These images demonstrate the biological relevance and clinical visibility of amygdalar degeneration in AD.

To better illustrate the rationale for selecting the amygdala as an intermediate phenotype in our framework, we provide a composite figure (Figure S1) combining mechanistic illustration, anatomical degeneration, and clinical imaging evidence[1]. Figure S1(a) presents a conceptual schematic of how AD-relevant modules (ADMs), derived from genotype data and protein interaction networks, are associated with clinical diagnosis (CN vs. AD) and mediated through amygdala-related iQTs. This highlights the functional integration of molecular and imaging data in the proposed framework. Figure S1(b) visualizes the process of amygdala atrophy, depicting a reduction in structural volume during the progression from cognitively normal status to AD. This reflects the widely reported vulnerability of the amygdala to early AD-related neurodegeneration, in line with neuropathological findings. Figure S1(c) provides representative coronal brain MRI slices comparing CN and AD individuals. The amygdala regions are highlighted and visibly reduced in volume in the AD group, supporting the clinical observability of the imaging phenotype. These neuroanatomical differences reinforce the amygdala's relevance in both diagnosis and mechanistic understanding of AD. These visual elements underscore the amygdala's role as a biologically meaningful and clinically interpretable imaging phenotype, justifying its use in identifying genetically mediated disease modules within our proposed framework.

S1.2 Genotyping data acquisition and processing

Genotyping data were obtained from the ADNI database (adni.loni.usc.edu). They were quality-controlled, imputed and combined as described in[2]. Briefly, genotyping was performed on all ADNI participants following manufacturer's protocol using blood genomic DNA samples and Illumina GWAS arrays (610-Quad, OmniExpress, or HumanOmni2.5–4v1)[3]. Quality control was performed in PLINK v1.90[4] using the following criteria: 1) call rate per marker $\geq$ 95%, 2) minor allele frequency (MAF) $\geq$ 5%, 3) Hardy Weinberg Equilibrium (HWE) test $P \geq$ 1.0E-6, and 4) call rate per participant $\geq$ 95%. Significant relatedness pairs with PI_HAT $>$ 0.45 were identified and thereafter one individual from each pair was randomly excluded (similar to the approach applied in[5]). Participants were then checked for gender and identity-by-descent before imputation to identify genotyping or coding error and to avoid the potential confounding effect due to the gender ambiguity or consanguinity such as sibling pairs. To restrict the studied participants to non-Hispanic Caucasians, we further performed population stratification using 988 subjects with known ancestry information from HapMap3 as reference data. We merged the ADNI and HapMap3 samples, and performed multidimensional scaling analysis using PLINK v1.90 with identity-by-state (IBS) pairwise distance matrix on the merged data to clustering samples in the principal component analysis (PCA) space. ADNI participants were identified as non-Hispanic Caucasians if: 1) they were clustered with HapMap3 CEU or TSI subjects as well as had self-reported race/ethnicity as "non-Hispanic/white", or 2) they were not clustered with any HapMap3 subjects while had self-reported race/ethnicity as "non-Hispanic/white". Haplotype patterns from the 1,000 Genomes Project reference panel were then applied to impute the SNPs that were not directly genotyped from arrays. 5,574,300 SNPs were obtained for all subjects involved in this work. To appropriately control for population stratification, we used PLINK v1.90 to generate the top four principal components to be included as covariates in our genetic association analyses.

S1.3 MRI data acquisition and processing

To investigate brain-associated MRI imaging phenotypes, we analyzed data from the ADNI database, including MRI scans from 1,515 participants[6]. Each participant underwent at least two baseline MP-RAGE scans at 1.5 T, following the ADNI MRI protocol[7]. The scans were processed using voxel-based morphometry (VBM), a widely employed automated MRI analysis method[8]. VBM analysis was conducted with SPM12 (http://www.fil.ion.ucl.ac.uk/spm/), which generated unmodulated, normalized grey matter (GM) density maps with a voxel resolution of $1 \times 1 \times 1$ mm, smoothed using a 10 mm full-width at half-maximum (FWHM) Gaussian kernel. For each participant, two independently processed maps were averaged to obtain a mean GM density map. Regional GM density values were extracted in the MNI space using the MarsBaR ROI toolbox[9]. A total of 86 phenotypes were defined (Table S1), corresponding to 43 anatomical regions, each represented by two measures: one for the left hemisphere and one for the right hemisphere. For instance, the left amygdala (LAmygdala) and right amygdala (RAmygdala) phenotypes represent the respective mean GM density values for these regions. In this study, particular attention was given to the phenotypes of the left and right amygdala, which were analyzed to investigate regional GM density differences.

S1.4 Network propagation to identify amygdala modules

To identify functional modules relevant to the amygdala, we applied network propagation on the amygdala-specific PPI network using the Hierarchical HotNet method[10], as shown in Figure 1 (c). Given a gene-scoring network $G = (V, E, w)$, where $n = |V|$ represents the number of nodes, $m = |E|$ represents the number of edges, and each node has an associated score $w(v_1), \ldots, w(v_n)$, a similarity matrix S is defined to quantify the similarity between pairs of nodes based on both the network topology and node scores. The similarity matrix S is calculated using a random walk with restart, where the similarity between nodes v_i and v_j is given by:

$$S = \beta \left(I - (1-\beta)AD^{-1}\right)^{-1} \cdot \mathrm{diag}(w(v_1), \ldots, w(v_n)), \tag{S1}$$

where A is the adjacency matrix, D is the diagonal degree matrix, β is the restart probability, and $w(v_1), \ldots, w(v_n)$ are the node scores. To construct the hierarchical structure, a directed graph $H_d = (V, E_d)$ is generated for each similarity threshold d, where an edge (v_j, v_i) exists if $s_{ij} \geq d$. The strongly connected components (SCCs) of H_d define clusters of nodes C_d. A series of clusterings $\{C_d\}_d$ is obtained and organized into a hierarchical dendrogram T. During the construction of the hierarchy, the statistical significance of each C_d within the T is evaluated

Table S1: Regions of interest (ROIs) and their corresponding phenotype IDs. Each phenotype is defined as the mean GM density of the ROI.

Phenotype ID	Region of Interest	Phenotype ID	Region of Interest
Amygdala	Amygdala	MidTempPole	Middle temporal pole
Angular	Angular gyrus	MidTemporal	Middle temporal gyrus
AntCingulate	Anterior cingulate	Olfactory	Olfactory gyrus
Fusiform	Fusiform gyrus	Parahipp	Parahippocampal gyrus
Heschl	Heschl's gyrus	PostCingulate	Posterior cingulate
Hippocampus	Hippocampus	Postcentral	Postcentral gyrus
InfFrontal_Oper	Inferior frontal operculum	Precentral	Precentral gyrus
InfFrontal_Triang	Inferior frontal triangularis	Precuneus	Precuneus
InfOrbFrontal	Inferior orbital frontal gyrus	Rectus	Rectus gyrus
InfParietal	Inferior parietal gyrus	Rolandic_Oper	Rolandic operculum
InfTemporal	Inferior temporal gyrus	Supfrontal	Superior frontal gyrus
Insula	Insula	SupOrbfrontal	Superior orbital frontal gyrus
Lingual	Lingual gyrus	SupParietal	Superior parietal gyrus
MedOrbFrontal	Medial orbital frontal gyrus	SupTempPole	Superior temporal pole
MedSupFrontal	Medial superior frontal gyrus	SupTemporal	Superior temporal gyrus
MidCingulate	Middle cingulate	SuppMotorArea	Supplementary motor area
MidFrontal	Middle frontal gyrus	Supramarg	Supramarginal gyrus
MidOrbFrontal	Middle orbital frontal gyrus	Thalamus	Thalamus

using a statistical permutation test to determine whether the observed SCCs represent significant structural features within the network. Specifically, for each similarity threshold d, we calculate the ratio of the observed maximum cluster size X_d to its expected size $\mathbb{E}[X_d]$ under the null hypothesis. The maximum of these ratios across all thresholds is then used to compute the corresponding P-value, which assesses the significance:

$$p = \Pr\left(\max_d \left(\frac{X_d}{\mathbb{E}[X_d]}\right) \geq y_{\max} \mid T\right), \tag{S2}$$

where $y_{\max}$ is the observed maximum ratio within the dendrogram T. Then the clustering $C_{d_{\max}}$ that corresponds to the threshold $d_{\max}$, which maximizes the ratio, is identified as the significant module. These modules represent regions of the network that are both highly connected and significantly elevated in node scores compared to what would be expected under random conditions. This process effectively extracts potentially biologically amygdala related modules from the amygdala specific network. In our study, d was set to 0, and statistical testing was conducted with 800 permutations and a minimum network size of 3.

S1.5 Evaluation of amygdala modules

The Genotype-Tissue Expression (GTEx) project[11] provides a comprehensive resource for studying tissue-specific gene expression and regulation across multiple human tissues. In this study, we utilized RNA-seq Gene TPMs (Transcripts Per Million) from GTEx V8 to evaluate the co-expression patterns of amygdala modules identified in our analysis.

S1.5.1 Assessment of modularity in the amygdala

To assess the modularity of the identified amygdala modules, we calculated the Pearson correlation coefficient (PCC) between all gene pairs within each module, based on the amygdala expression data. The module-level PCC was defined as the average PCC of all gene pairs in that module. Formally, for each module M_i containing g_i genes, we first computed the gene-gene correlation matrix R_i using Pearson's correlation. Each element r_{jk} of R_i represents the PCC between gene j and gene k. The module-level PCC $\overline{r}_{M_i}$ was then calculated as:

$$\overline{r}_{M_i} = \frac{1}{\binom{g_i}{2}} \sum_{j<k} |r_{jk}|, \tag{S3}$$

where $\binom{g_i}{2}$ denotes the number of unique gene pairs in module M_i. To determine whether the observed co-expression within each module was stronger than expected by chance, we generated 1,000 random modules for each identified amygdala module, each with the same number of genes

as the original module. The module-level PCC for each random module was computed using the same formula S3. A paired t-test was then performed to compare the module-level PCC of the identified amygdala module against the distribution of the module-level PCCs derived from the corresponding 1,000 random modules. A significant p-value would suggest that the identified amygdala modules have a statistically significant modularity, which is unlikely to occur by chance.

S1.5.2 Validation of tissue specificity

To further confirm the specificity of the identified amygdala modules, we compared their co-expression levels in the amygdala to those in other tissues, including the hippocampus, brain cortex, and whole blood. Specifically, for each module M_i, we obtained the module-level PCC in these tissues by repeating the above calculation using their respective expression data. Let $\mathcal{C}_{amygdala} = \{\overline{r}_{M_1}, \overline{r}_{M_2}, \ldots, \overline{r}_{M_n}\}$ represent the set of $\overline{r}_{M_i}$ values for the amygdala using equation S3. Similarly, for other tissues such as the hippocampus, cortex, and whole blood, we obtained the corresponding sets as $\mathcal{C}_{hippo}$, $\mathcal{C}_{cortex}$, and $\mathcal{C}_{blood}$, respectively. A paired t-test was then performed to compare the module-level PCCs between the amygdala set $\mathcal{C}_{amygdala}$ and each of the other tissue sets $\mathcal{C}_{hippo}$, $\mathcal{C}_{cortex}$, and $\mathcal{C}_{blood}$. A significant P-value would indicate that the modules exhibit higher co-expression in the amygdala than in the other tissues, suggesting tissue specificity.

S1.6 Evaluation of ADMs

To assess the modularity of each identified ADM using the GTEx V8 RNA-seq Gene TPMs from the amygdala tissue, we conducted a two-step procedure.

S1.6.1 Step 1: Visualization and preliminary assessment

To provide a visual comparison, we constructed a correlation heatmap using the genes from the ADM along with a randomly selected module from the 1,000 generated random modules for each ADM. Darker regions within the ADM portion of the heatmap were indicative of higher internal correlation among the module's genes compared to the randomly selected module, suggesting a stronger modular structure for the ADM.

S1.6.2 Step 2: Statistical evaluation via permutation testing

To determine the statistical significance of the observed modularity, we conducted a permutation test. For each ADM, we calculated the module-level PCC for the ADM (denoted as the "observed" module) and for each of the 1,000 random modules. The P-value was defined as the proportion of random modules whose module-level PCC met or exceeded that of the observed ADM:

$$p = \frac{\sum_{i=1}^{1000} \mathbb{I}(\overline{r}_{M_i^{\text{random}}} \geq \overline{r}_{M_i^{\text{observed}}})}{1000}, \tag{S4}$$

where $\overline{r}_{M_i^{\text{observed}}}$ is the module-level PCC for the identified (observed) module, and $\mathbb{I}(\cdot)$ is an indicator function that equals 1 if the condition inside is true, and 0 otherwise. A P-value less than 0.05 suggests that the observed ADM has significantly higher modularity compared to random modules.

S2 Supplementary Results

S2.1 GWAS and GSA of amygdala QTs

GWAS of amygdala phenotypes: We conducted SNP-level and gene-level analyses on the left and right amygdala to assess the association between genetic variants and normalized amygdala volume in 1,515 ADNI subjects. An overview of the data is provided in Table 1, which includes summary statistics for all diagnostic groups (CN, SMC, EMCI, LMCI, and AD). Age, education level, and normalized amygdala volume (both left and right) showed significant differences across the five diagnostic groups ($P < 0.001$). In the SNP-level analysis, we tested the association of 5,574,300 SNPs with normalized amygdala volume, adjusting for covariates. Figure S2 displays significant SNPs for both the left and right amygdala. Using a genome-wide significance threshold of 5.0E-8, we identified 10 variants in the left amygdala and seven in the right. The top SNP was rs429358 (P = 6.93E-14) in the *APOE* gene, followed by rs12721051 (P = 9.71E-11), rs56131196 (P = 9.71E-11), rs4420638 (P = 9.71E-11), and rs438811 (P = 1.65E-09) in the *APOE* or *APOC1*

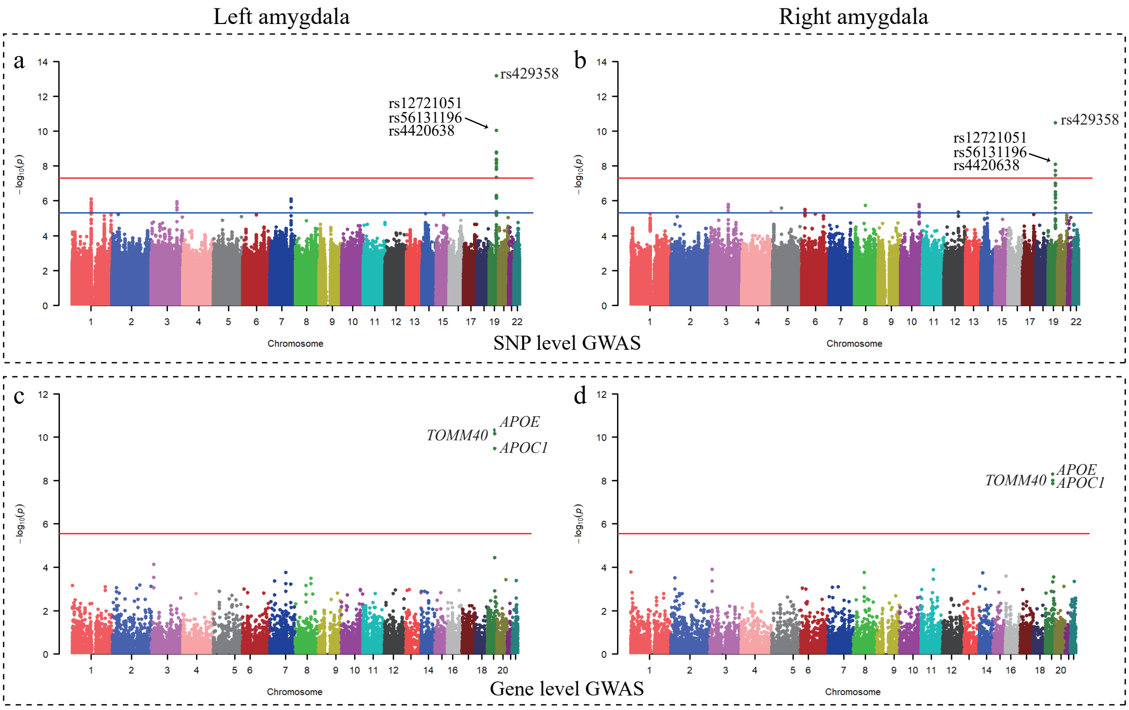

Figure S2: GWAS Manhattan plots of the amygdala at both the SNP and gene levels. (**a**) and (**b**) show SNP-level associations for the left and right amygdala, respectively, with blue and red lines indicating P-value thresholds of 5E-6 and 5E-8. (**c**) and (**d**) demonstrate gene-level associations for the left and right amygdala, respectively, with the red line marking a P-value threshold of 2.76E-6.

gene region. The top four significant variants for the right amygdala were the same as those identified for the left amygdala.

Gene set analysis of amygdala phenotypes: In the gene set analysis, after applying Bonferroni correction with a corrected P-value threshold of 2.76E-6, three genes were identified as significantly associated with amygdala volume: *APOE* ($P = 4.82$E-11), *TOMM40* ($P = 7.12$E-11), and *APOC1* ($P = 3.44$E-10).

The *APOE* ϵ4 allele, encoded by rs429358, markedly escalates the lifetime risk of developing AD, significantly impacting both the risk and the age of onset[12,13]. A study reveals that the influence of the *APOE* genotype on the amygdala is primarily observed in young-old patients with MCI and AD, with the most significant effects seen in the basolateral, centromedial, and lateral nuclei of the right amygdala, while the basomedial nucleus is less affected[14]. The *APOC1* gene plays a significant role in lipid metabolism and is linked to both Type 2 Diabetes and AD through the rs12721051 identified in GWAS[15]. The association of rs12721051 with AD is further elucidated by its relation to the *APOE* gene and the rs429358, highlighting a complex genetic network involving *APOC1* and *APOE* in AD pathogenesis with the *APOE* ϵ4 allele[16]. *TOMM40* was also reported to be specifically associated with right amygdala volume[6].

Table S2: Significant SNPs identified in the SNP-level GWAS. The * presents both significant in the left and right amygdala.

Left Amygdala (20 variants)			Right Amygdala (seven variants)		
SNP	Gene	P-value	SNP	Gene	P-value
*rs429358	*APOE*	6.93E-14	*rs429358	*APOE*	3.33E-11
*rs12721051	*APOC1*	9.71E-11	*rs12721051	APOC1	8.24E-09
*rs56131196	*APOC1*	9.71E-11	*rs56131196	*APOC1*	8.24E-09
*rs4420638	*APOC1*	9.71E-11	*rs4420638	*APOC1*	8.24E-09
rs438811	*APOC1*	1.65E-09	*rs769449	*APOE*	1.95E-08
*rs769449	APOE	1.75E-09	*rs10414043	*APOC1*	3.48E-08
rs483082	*APOC1*	1.80E-09	*rs7256200	*APOC1*	3.48E-08
rs5117	*APOC1*	1.82E-09			
rs59007384	*TOMM40*	1.90E-09			
rs283812	*NECTIN2*	4.21E-09			
*rs10414043	*APOC1*	5.05E-09			
*rs7256200	*APOC1*	5.05E-09			
rs184017	*TOMM40*	6.81E-09			
rs157581	*TOMM40*	6.81E-09			
rs157582	*TOMM40*	6.81E-09			
rs6857	*NECTIN2*	7.84E-09			
rs10119	*TOMM40*	1.18E-08			
rs283811	*NECTIN2*	1.54E-08			
rs283815	*NECTIN2*	1.56E-08			
rs73052335	*APOC1*	4.61E-08			

Table S3: Significant genes identified in the GSA

Left amygdala		Right amygdala	
Gene	P-value	Gene	P-value
APOE	4.82E-11	*APOE*	5.12E-9
TOMM40	7.12E-11	*TOMM40*	1.01E-08
APOC1	3.44E-10	*APOC1*	1.37E-08

S2.2 Functional annotation of ADMs

S2.2.1 GO-BP gene-concept network

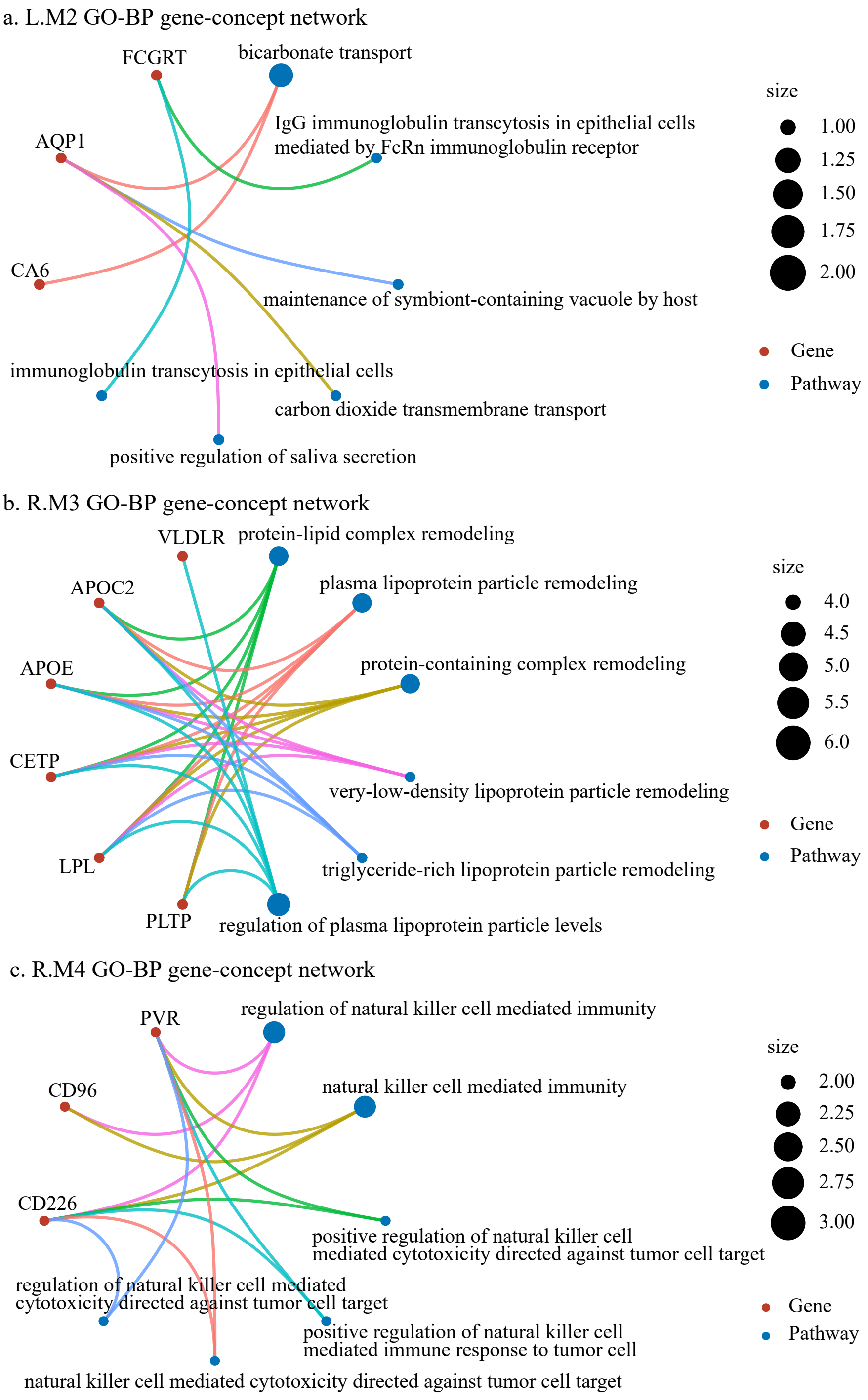

Figure S3: Functional enrichment of ADMs, including L.M2, R.M3, and R.M4.

S2.2.2 Significantly enriched pathways of L.M1

Table S4: GO-BP enriched pathways of L.M1

ID	Description	qvalue	geneID
GO:0140056	organelle localization by membrane tethering	5.71E-08	RAB3IP, VPS33A, VPS18, CEP72, VTI1B, RAB3D, VAMP8, STX7, PCM1, RAB8A
GO:0022406	membrane docking	5.71E-08	RAB3IP, VPS33A, VPS18, CEP72, VTI1B, RAB3D, VAMP8, STX7, PCM1, RAB8A
GO:0048278	vesicle docking	1.20E-05	VPS33A, VPS18, VTI1B, RAB3D, STX7, RAB8A
GO:0140029	exocytic process	3.68E-05	VPS33A, VPS18, VTI1B, RAB3D, VAMP8, RAB8A
GO:0006904	vesicle docking involved in exocytosis	3.68E-05	VPS33A, VPS18, VTI1B, RAB3D, RAB8A
GO:0006906	vesicle fusion	8.51E-05	VTI1A, VPS18, VTI1B, VAMP8, STX7, RAB8A
GO:0090174	organelle membrane fusion	1.03E-04	VTI1A, VPS18, VTI1B, VAMP8, STX7, RAB8A
GO:0060271	cilium assembly	1.84E-04	BBS5, TTC8, RAB3IP, CEP72, BBS9, IFT88, PCM1, RAB8A, BBS1
GO:0044782	cilium organization	2.50E-04	BBS5, TTC8, RAB3IP, CEP72, BBS9, IFT88, PCM1, RAB8A, BBS1
GO:0048284	organelle fusion	3.46E-04	VTI1A, VPS18, VTI1B, VAMP8, STX7, RAB8A
GO:0016050	vesicle organization	6.01E-04	VTI1A, VPS18, SCAP, VTI1B, VAMP8, STX7, RAB8A, INSIG1
GO:0061025	membrane fusion	8.51E-04	VTI1A, VPS18, VTI1B, VAMP8, STX7, RAB8A
GO:0048193	Golgi vesicle transport	1.02E-03	VTI1A, SCAP, VTI1B, VAMP8, RAB8A, INSIG1, GOLGB1, BBS1
GO:1905515	non-motile cilium assembly	1.57E-03	TTC8, IFT88, PCM1, BBS1
GO:0010894	negative regulation of steroid biosynthetic process	4.94E-03	SCAP, INSIG1, APOE
GO:0007034	vacuolar transport	4.94E-03	VTI1A, MON1A, VPS33A, VPS18, VTI1B
GO:0010977	negative regulation of neuron projection development	5.09E-03	RTN4RL1, LINGO1, RTN4R, OMG, APOE
GO:0045939	negative regulation of steroid metabolic process	5.09E-03	SCAP, INSIG1, APOE
GO:0071539	protein localization to centrosome	5.33E-03	CEP72, PCM1, GOLGB1
GO:0042744	hydrogen peroxide catabolic process	5.57E-03	HP, HBG2, HBB
GO:1905508	protein localization to microtubule organizing center	5.83E-03	CEP72, PCM1, GOLGB1
GO:0072659	protein localization to plasma membrane	7.89E-03	VTI1B, RAB3D, VAMP8, STX7, RAB8A, BBS1
GO:0006623	protein targeting to vacuole	8.84E-03	VTI1A, MON1A, VTI1B
GO:0050768	negative regulation of neurogenesis	8.84E-03	RTN4RL1, LINGO1, RTN4R, PCM1, OMG, APOE

ID	Description	qvalue	geneID
GO:0031345	negative regulation of cell projection organization	8.84E-03	RTN4RL1, LINGO1, RTN4R, OMG, APOE
GO:0048280	vesicle fusion with Golgi apparatus	8.84E-03	VTI1A, VTI1B
GO:0098869	cellular oxidant detoxification	8.84E-03	HP, HBG2, HBB, APOE
GO:0006892	post-Golgi vesicle-mediated transport	8.84E-03	VTI1A, VTI1B, VAMP8, BBS1
GO:0045541	negative regulation of cholesterol biosynthetic process	9.26E-03	SCAP, APOE
GO:0090206	negative regulation of cholesterol metabolic process	9.26E-03	SCAP, APOE
GO:0097267	omega-hydroxylase P450 pathway	9.26E-03	CYP2C9, CYP2C8
GO:0106119	negative regulation of sterol biosynthetic process	9.26E-03	SCAP, APOE
GO:0051961	negative regulation of nervous system development	1.00E-02	RTN4RL1, LINGO1, RTN4R, PCM1, OMG, APOE
GO:0006888	endoplasmic reticulum to Golgi vesicle-mediated transport	1.00E-02	VTI1A, SCAP, VTI1B, INSIG1, GOLGB1
GO:1990748	cellular detoxification	1.00E-02	HP, HBG2, HBB, APOE
GO:0097237	cellular response to toxic substance	1.26E-02	HP, HBG2, HBB, APOE
GO:0072666	establishment of protein localization to vacuole	1.26E-02	VTI1A, MON1A, VTI1B
GO:1990778	protein localization to cell periphery	1.26E-02	VTI1B, RAB3D, VAMP8, STX7, RAB8A, BBS1
GO:0010721	negative regulation of cell development	1.26E-02	RTN4RL1, LINGO1, RTN4R, PCM1, OMG, APOE
GO:0072698	protein localization to microtubule cytoskeleton	1.26E-02	CEP72, PCM1, GOLGB1
GO:0045665	negative regulation of neuron differentiation	1.26E-02	RTN4RL1, LINGO1, RTN4R, OMG, APOE
GO:0042743	hydrogen peroxide metabolic process	1.45E-02	HP, HBG2, HBB
GO:0044380	protein localization to cytoskeleton	1.45E-02	CEP72, PCM1, GOLGB1
GO:0031638	zymogen activation	1.46E-02	C1RL, PRSS12, HP
GO:0051055	negative regulation of lipid biosynthetic process	1.46E-02	SCAP, INSIG1, APOE
GO:0031102	neuron projection regeneration	1.50E-02	RTN4RL1, RTN4R, OMG
GO:0098754	detoxification	1.51E-02	HP, HBG2, HBB, APOE
GO:0032933	SREBP signaling pathway	1.51E-02	SCAP, INSIG1
GO:0048681	negative regulation of axon regeneration	1.51E-02	RTN4RL1, RTN4R
GO:0015671	oxygen transport	1.61E-02	HBG2, HBB
GO:0070571	negative regulation of neuron projection regeneration	1.61E-02	RTN4RL1, RTN4R
GO:0071501	cellular response to sterol depletion	1.61E-02	SCAP, INSIG1
GO:0090110	COPII-coated vesicle cargo loading	1.61E-02	SCAP, INSIG1

ID	Description	qvalue	geneID
GO:0072665	protein localization to vacuole	1.90E-02	VTI1A, MON1A, VTI1B
GO:0008203	cholesterol metabolic process	1.90E-02	SCAP, INSIG1, CYP2C9, APOE
GO:0006896	Golgi to vacuole transport	1.90E-02	VTI1A, VTI1B
GO:0006991	response to sterol depletion	1.90E-02	SCAP, INSIG1
GO:1902931	negative regulation of alcohol biosynthetic process	1.90E-02	SCAP, APOE
GO:0050771	negative regulation of axonogenesis	1.92E-02	LINGO1, RTN4R, OMG
GO:0034375	high-density lipoprotein particle remodeling	2.06E-02	PLTP, APOE
GO:1902652	secondary alcohol metabolic process	2.15E-02	SCAP, INSIG1, CYP2C9, APOE
GO:0070989	oxidative demethylation	2.17E-02	CYP2C9, CYP2C8
GO:0006695	cholesterol biosynthetic process	2.17E-02	SCAP, INSIG1, APOE
GO:1902653	secondary alcohol biosynthetic process	2.17E-02	SCAP, INSIG1, APOE
GO:0016125	sterol metabolic process	2.19E-02	SCAP, INSIG1, CYP2C9, APOE
GO:0010875	positive regulation of cholesterol efflux	2.19E-02	PLTP, APOE
GO:0015669	gas transport	2.19E-02	HBG2, HBB
GO:0019373	epoxygenase P450 pathway	2.19E-02	CYP2C9, CYP2C8
GO:0022038	corpus callosum development	2.19E-02	RTN4RL1, RTN4R
GO:0035493	SNARE complex assembly	2.19E-02	VPS18, VAMP8
GO:0031503	protein-containing complex localization	2.30E-02	CEP72, STX7, IFT88, PCM1, RAB8A
GO:0006633	fatty acid biosynthetic process	2.31E-02	SCAP, INSIG1, CYP2C9, CYP2C8
GO:0051604	protein maturation	2.31E-02	HSCB, C1RL, ZMPSTE24, PRSS12, HP
GO:0016126	sterol biosynthetic process	2.34E-02	SCAP, INSIG1, APOE
GO:0032799	low-density lipoprotein receptor particle metabolic process	2.70E-02	SCAP, APOE
GO:0050770	regulation of axonogenesis	2.86E-02	LINGO1, RTN4R, OMG, APOE
GO:0042738	exogenous drug catabolic process	2.86E-02	CYP2C9, CYP2C8
GO:0032400	melanosome localization	2.86E-02	BBS5, VPS33A
GO:1903035	negative regulation of response to wounding	2.86E-02	RTN4RL1, RTN4R, APOE
GO:0042737	drug catabolic process	2.86E-02	CYP2C9, CYP2C8
GO:0051875	pigment granule localization	2.86E-02	BBS5, VPS33A
GO:0051668	localization within membrane	2.86E-02	SCAP, INSIG1, APOE
GO:0097711	ciliary basal body-plasma membrane docking	2.86E-02	RAB3IP, CEP72, PCM1
GO:0035459	vesicle cargo loading	2.86E-02	SCAP, INSIG1
GO:0010189	vitamin E biosynthetic process	2.86E-02	PLTP
GO:0015904	tetracycline transmembrane transport	2.86E-02	MFSD10

ID	Description	qvalue	geneID
GO:0043979	histone H2B-K5 acetylation	2.86E-02	ZMPSTE24
GO:0071831	intermediate-density lipoprotein particle clearance	2.86E-02	APOE
GO:0090731	cellular response to very-low-density lipoprotein particle stimulus	2.86E-02	APOE
GO:0150200	regulation of transport across blood-brain barrier	2.86E-02	APOE
GO:0150201	positive regulation of transport across blood-brain barrier	2.86E-02	APOE
GO:1901303	negative regulation of cargo loading into COPII-coated vesicle	2.86E-02	INSIG1
GO:1903000	regulation of lipid transport across blood-brain barrier	2.86E-02	APOE
GO:1903002	positive regulation of lipid transport across blood-brain barrier	2.86E-02	APOE
GO:1905853	regulation of heparan sulfate binding	2.86E-02	APOE
GO:1905855	positive regulation of heparan sulfate binding	2.86E-02	APOE
GO:1905858	regulation of heparan sulfate proteoglycan binding	2.86E-02	APOE
GO:1905860	positive regulation of heparan sulfate proteoglycan binding	2.86E-02	APOE
GO:1905890	regulation of cellular response to very-low-density lipoprotein particle stimulus	2.86E-02	APOE
GO:2000730	regulation of termination of RNA polymerase I transcription	2.86E-02	ZMPSTE24
GO:0008202	steroid metabolic process	2.86E-02	SCAP, INSIG1, CYP2C9, CYP2C8, APOE
GO:0010771	negative regulation of cell morphogenesis involved in differentiation	2.86E-02	LINGO1, RTN4R, OMG
GO:0045833	negative regulation of lipid metabolic process	2.86E-02	SCAP, INSIG1, APOE
GO:0050810	regulation of steroid biosynthetic process	2.86E-02	SCAP, INSIG1, APOE
GO:0032373	positive regulation of sterol transport	3.01E-02	PLTP, APOE
GO:0032376	positive regulation of cholesterol transport	3.01E-02	PLTP, APOE
GO:0048679	regulation of axon regeneration	3.01E-02	RTN4RL1, RTN4R
GO:1903076	regulation of protein localization to plasma membrane	3.08E-02	VTI1B, VAMP8, STX7
GO:0062014	negative regulation of small molecule metabolic process	3.28E-02	SCAP, INSIG1, APOE

ID	Description	qvalue	geneID
GO:0034368	protein-lipid complex remodeling	3.28E-02	PLTP, APOE
GO:0034369	plasma lipoprotein particle remodeling	3.28E-02	PLTP, APOE
GO:0045940	positive regulation of steroid metabolic process	3.28E-02	SCAP, APOE
GO:0008210	estrogen metabolic process	3.40E-02	CYP2C9, CYP2C8
GO:0034367	protein-containing complex remodeling	3.40E-02	PLTP, APOE
GO:0070570	regulation of neuron projection regeneration	3.40E-02	RTN4RL1, RTN4R
GO:0017157	regulation of exocytosis	3.53E-02	VPS18, RAB3D, VAMP8, RAB8A
GO:0016485	protein processing	3.56E-02	C1RL, ZMPSTE24, PRSS12, HP
GO:0006891	intra-Golgi vesicle-mediated transport	3.69E-02	VTI1A, VTI1B
GO:0042759	long-chain fatty acid biosynthetic process	3.69E-02	CYP2C9, CYP2C8
GO:0046394	carboxylic acid biosynthetic process	3.69E-02	SCAP, INSIG1, CYP2C9, CYP2C8, BHMT
GO:0016053	organic acid biosynthetic process	3.70E-02	SCAP, INSIG1, CYP2C9, CYP2C8, BHMT
GO:0006066	alcohol metabolic process	4.29E-02	SCAP, INSIG1, CYP2C9, CYP2C8, APOE
GO:1904375	regulation of protein localization to cell periphery	4.29E-02	VTI1B, VAMP8, STX7
GO:0010877	lipid transport involved in lipid storage	4.29E-02	APOE
GO:0031106	septin ring organization	4.29E-02	RTKN
GO:0033566	gamma-tubulin complex localization	4.29E-02	CEP72
GO:0036316	SREBP-SCAP complex retention in endoplasmic reticulum	4.29E-02	INSIG1
GO:0043007	maintenance of rDNA	4.29E-02	ZMPSTE24
GO:0048560	establishment of anatomical structure orientation	4.29E-02	TTC8
GO:0050666	regulation of homocysteine metabolic process	4.29E-02	BHMT
GO:1901301	regulation of cargo loading into COPII-coated vesicle	4.29E-02	INSIG1
GO:1905793	protein localization to pericentriolar material	4.29E-02	GOLGB1
GO:2000296	negative regulation of hydrogen peroxide catabolic process	4.29E-02	HP
GO:0006721	terpenoid metabolic process	4.29E-02	CYP2C9, CYP2C8, APOE
GO:0072330	monocarboxylic acid biosynthetic process	4.35E-02	SCAP, INSIG1, CYP2C9, CYP2C8
GO:0035735	intraciliary transport involved in cilium assembly	4.42E-02	IFT88, PCM1
GO:0097352	autophagosome maturation	4.42E-02	VPS33A, VAMP8
GO:0009636	response to toxic substance	4.50E-02	HP, HBG2, HBB, APOE
GO:0019218	regulation of steroid metabolic process	4.81E-02	SCAP, INSIG1, APOE

ID	Description	qvalue	geneID
GO:1901617	organic hydroxy compound biosynthetic process	4.95E-02	SCAP, PLTP, INSIG1, APOE

Table S5: KEGG enriched pathways of L.M1

ID	Description	qvalue	geneID
hsa04130	SNARE interactions in vesicular transport	1.48E-04	VTI1A, VTI1B, VAMP8, STX7

Table S6: DO enriched pathways of L.M1

ID	Description	qvalue	geneID
DOID:1935	Bardet-Biedl syndrome	1.79E-04	BBS5, TTC8, BBS9, BBS1
DOID:3393	coronary artery disease	7.70E-04	SCAP, CD163, VAMP8, PLTP, INSIG1, HP, CYP2C9, CYP2C8, APOE
DOID:0050737	autosomal recessive disease	3.16E-03	BBS5, TTC8, VPS33A, BBS9, ZMPSTE24, HBG2, HBB, BBS1
DOID:4990	essential tremor	3.50E-03	LINGO1, CYP2C9, CYP2C8
DOID:5844	myocardial infarction	4.58E-03	SCAP, CD163, VAMP8, HP, CYP2C9, CYP2C8, APOE
DOID:13832	patent ductus arteriosus	1.15E-02	CYP2C9, CYP2C8
DOID:9279	hyperhomocysteinemia	1.31E-02	BHMT, APOE
DOID:1803	neuritis	3.16E-02	RTKN, APOE
DOID:1682	congenital heart disease	3.79E-02	CYP2C9, CYP2C8, APOE

S2.2.3 Significantly enriched pathways of L.M2

Table S7: GO-BP enriched pathways of L.M2

ID	Description	qvalue	geneID
GO:0015701	bicarbonate transport	2.90E-02	CA6, AQP1
GO:0002416	IgG immunoglobulin transcytosis in epithelial cells mediated by FcRn immunoglobulin receptor	2.90E-02	FCGRT
GO:0085018	maintenance of symbiont-containing vacuole by host	2.90E-02	AQP1
GO:0035378	carbon dioxide transmembrane transport	2.90E-02	AQP1
GO:0046878	positive regulation of saliva secretion	2.90E-02	AQP1
GO:0002414	immunoglobulin transcytosis in epithelial cells	2.90E-02	FCGRT
GO:0030185	nitric oxide transport	2.90E-02	AQP1
GO:0035377	transepithelial water transport	2.90E-02	AQP1
GO:0044313	protein K6-linked deubiquitination	2.90E-02	USP30
GO:0071288	cellular response to mercury ion	2.90E-02	AQP1
GO:0033326	cerebrospinal fluid secretion	2.90E-02	AQP1

ID	Description	qvalue	geneID
GO:0015670	carbon dioxide transport	2.90E-02	AQP1
GO:0030950	establishment or maintenance of actin cytoskeleton polarity	2.90E-02	AQP1
GO:0002860	positive regulation of natural killer cell mediated cytotoxicity directed against tumor cell target	2.90E-02	CD226
GO:0003097	renal water transport	2.90E-02	AQP1
GO:0002857	positive regulation of natural killer cell mediated immune response to tumor cell	2.90E-02	CD226
GO:0009992	cellular water homeostasis	2.90E-02	AQP1
GO:0015793	glycerol transport	2.90E-02	AQP1
GO:0046877	regulation of saliva secretion	2.90E-02	AQP1
GO:0060369	positive regulation of Fc receptor mediated stimulatory signaling pathway	2.90E-02	CD226
GO:1901525	negative regulation of mitophagy	2.90E-02	USP30
GO:0002420	natural killer cell mediated cytotoxicity directed against tumor cell target	2.90E-02	CD226
GO:0002729	positive regulation of natural killer cell cytokine production	2.90E-02	CD226
GO:0002858	regulation of natural killer cell mediated cytotoxicity directed against tumor cell target	2.90E-02	CD226
GO:0002370	natural killer cell cytokine production	2.90E-02	CD226
GO:0002423	natural killer cell mediated immune response to tumor cell	2.90E-02	CD226
GO:0002727	regulation of natural killer cell cytokine production	2.90E-02	CD226
GO:0002855	regulation of natural killer cell mediated immune response to tumor cell	2.90E-02	CD226
GO:0030952	establishment or maintenance of cytoskeleton polarity	2.90E-02	AQP1
GO:0042045	epithelial fluid transport	2.90E-02	AQP1
GO:1903147	negative regulation of autophagy of mitochondrion	2.90E-02	USP30
GO:0015696	ammonium transport	2.90E-02	AQP1
GO:0035871	protein K11-linked deubiquitination	2.90E-02	USP30
GO:0072488	ammonium transmembrane transport	2.90E-02	AQP1
GO:0060368	regulation of Fc receptor mediated stimulatory signaling pathway	2.90E-02	CD226
GO:0021670	lateral ventricle development	2.90E-02	AQP1
GO:0030157	pancreatic juice secretion	2.90E-02	AQP1

ID	Description	qvalue	geneID
GO:0046541	saliva secretion	2.90E-02	AQP1
GO:0046689	response to mercury ion	2.90E-02	AQP1
GO:0071472	cellular response to salt stress	2.90E-02	AQP1
GO:0002836	positive regulation of response to tumor cell	2.90E-02	CD226
GO:0002839	positive regulation of immune response to tumor cell	2.90E-02	CD226
GO:0015791	polyol transport	2.90E-02	AQP1
GO:0019755	one-carbon compound transport	2.90E-02	AQP1
GO:0060456	positive regulation of digestive system process	2.90E-02	AQP1
GO:0050862	positive regulation of T cell receptor signaling pathway	3.20E-02	CD226
GO:0071474	cellular hyperosmotic response	3.20E-02	AQP1
GO:0002834	regulation of response to tumor cell	3.21E-02	CD226
GO:0002837	regulation of immune response to tumor cell	3.21E-02	CD226
GO:0071732	cellular response to nitric oxide	3.21E-02	AQP1
GO:1901524	regulation of mitophagy	3.54E-02	USP30
GO:1902170	cellular response to reactive nitrogen species	3.58E-02	AQP1
GO:0002418	immune response to tumor cell	3.58E-02	CD226
GO:0015669	gas transport	3.58E-02	AQP1
GO:0045056	transcytosis	3.58E-02	FCGRT
GO:0071731	response to nitric oxide	3.58E-02	AQP1
GO:0006833	water transport	3.69E-02	AQP1
GO:0033005	positive regulation of mast cell activation	3.80E-02	CD226
GO:0032469	endoplasmic reticulum calcium ion homeostasis	3.91E-02	KCTD17
GO:0045724	positive regulation of cilium assembly	3.91E-02	KCTD17
GO:0045954	positive regulation of natural killer cell mediated cytotoxicity	3.91E-02	CD226
GO:0000423	mitophagy	3.91E-02	USP30
GO:0039529	RIG-I signaling pathway	3.91E-02	CLPB
GO:0050857	positive regulation of antigen receptor-mediated signaling pathway	3.91E-02	CD226
GO:0002347	response to tumor cell	3.94E-02	CD226
GO:0070633	transepithelial transport	3.94E-02	AQP1
GO:0008053	mitochondrial fusion	3.94E-02	USP30
GO:0009651	response to salt stress	3.94E-02	AQP1
GO:0006972	hyperosmotic response	3.94E-02	AQP1
GO:0042044	fluid transport	3.94E-02	AQP1
GO:0071280	cellular response to copper ion	3.94E-02	AQP1
GO:0002717	positive regulation of natural killer cell mediated immunity	4.02E-02	CD226

ID	Description	qvalue	geneID
GO:0006884	cell volume homeostasis	4.05E-02	AQP1
GO:0019934	cGMP-mediated signaling	4.05E-02	AQP1
GO:0039528	cytoplasmic pattern recognition receptor signaling pathway in response to virus	4.07E-02	CLPB
GO:0071549	cellular response to dexamethasone stimulus	4.07E-02	AQP1
GO:0021591	ventricular system development	4.15E-02	AQP1
GO:0016242	negative regulation of macroautophagy	4.35E-02	USP30
GO:0001580	detection of chemical stimulus involved in sensory perception of bitter taste	4.54E-02	CA6
GO:0003091	renal water homeostasis	4.55E-02	AQP1
GO:0032941	secretion by tissue	4.55E-02	AQP1
GO:0002714	positive regulation of B cell mediated immunity	4.60E-02	CD226
GO:0002891	positive regulation of immunoglobulin mediated immune response	4.60E-02	CD226
GO:0006730	one-carbon metabolic process	4.60E-02	CA6
GO:0042269	regulation of natural killer cell mediated cytotoxicity	4.60E-02	CD226
GO:0050913	sensory perception of bitter taste	4.60E-02	CA6
GO:0071548	response to dexamethasone	4.60E-02	AQP1
GO:0044058	regulation of digestive system process	4.60E-02	AQP1
GO:0050912	detection of chemical stimulus involved in sensory perception of taste	4.60E-02	CA6
GO:0071470	cellular response to osmotic stress	4.60E-02	AQP1
GO:0046688	response to copper ion	4.60E-02	AQP1
GO:0050856	regulation of T cell receptor signaling pathway	4.60E-02	CD226
GO:1903146	regulation of autophagy of mitochondrion	4.60E-02	USP30
GO:0033003	regulation of mast cell activation	4.66E-02	CD226
GO:0002715	regulation of natural killer cell mediated immunity	4.71E-02	CD226
GO:0001819	positive regulation of cytokine production	4.81E-02	CLPB, CD226
GO:0008089	anterograde axonal transport	4.92E-02	ARL8A
GO:0006979	response to oxidative stress	4.93E-02	NDUFA6, AQP1

Table S8: DO enriched pathways of L.M2

ID	Description	qvalue	geneID
DOID:12215	oligohydramnios	2.42E-02	AQP1
DOID:3304	germinoma	2.42E-02	AQP1
DOID:4226	endometrial stromal sarcoma	2.42E-02	CD226
DOID:9849	Meniere's disease	2.42E-02	AQP1
DOID:11396	pulmonary edema	2.42E-02	AQP1
DOID:3426	vestibular disease	2.42E-02	AQP1
DOID:4724	brain edema	2.42E-02	AQP1
DOID:9847	peripheral vertigo	2.42E-02	AQP1
DOID:9848	endolymphatic hydrops	2.42E-02	AQP1
DOID:6419	tetralogy of Fallot	2.42E-02	LPP
DOID:11949	Creutzfeldt-Jakob disease	2.42E-02	AQP1
DOID:5166	endometrial stromal tumor	2.42E-02	CD226
DOID:5241	hemangioblastoma	2.42E-02	AQP1
DOID:171	neuroectodermal tumor	2.42E-02	AQP1
DOID:12132	Wegener's granulomatosis	2.52E-02	CD226
DOID:4539	labyrinthine disease	2.58E-02	AQP1
DOID:649	prion disease	2.58E-02	AQP1
DOID:780	placenta disease	2.93E-02	AQP1
DOID:12177	common variable immunodeficiency	2.93E-02	FCGRT
DOID:2583	agammaglobulinemia	2.93E-02	FCGRT
DOID:620	blood protein disease	2.93E-02	FCGRT
DOID:2115	B cell deficiency	2.98E-02	FCGRT
DOID:4928	intrahepatic cholangiocarcinoma	3.06E-02	AQP1
DOID:255	hemangioma	3.06E-02	AQP1
DOID:12217	Lewy body dementia	3.20E-02	AQP1
DOID:1319	brain cancer	3.20E-02	AQP1
DOID:345	uterine disease	3.52E-02	AQP1
DOID:1070	primary open angle glaucoma	3.54E-02	AQP1
DOID:11162	respiratory failure	3.75E-02	AQP1
DOID:1682	congenital heart disease	3.82E-02	LPP
DOID:1067	open-angle glaucoma	3.89E-02	AQP1
DOID:3068	glioblastoma multiforme	4.23E-02	AQP1
DOID:2952	inner ear disease	4.23E-02	AQP1
DOID:6364	migraine	4.23E-02	AQP1

S2.2.4 Significantly enriched pathways of R.M3

Table S9: GO-BP enriched pathways of R.M3

ID	Description	qvalue	geneID
GO:0034368	protein-lipid complex remodeling	5.79E-08	PLTP, LPL, CETP, APOE, APOC2
GO:0034369	plasma lipoprotein particle remodeling	5.79E-08	PLTP, LPL, CETP, APOE, APOC2
GO:0034367	protein-containing complex remodeling	5.79E-08	PLTP, LPL, CETP, APOE, APOC2
GO:0034372	very-low-density lipoprotein particle remodeling	7.17E-08	LPL, CETP, APOE, APOC2
GO:0034370	triglyceride-rich lipoprotein particle remodeling	1.24E-07	LPL, CETP, APOE, APOC2

ID	Description	qvalue	geneID
GO:0097006	regulation of plasma lipoprotein particle levels	2.09E-07	VLDLR, PLTP, LPL, CETP, APOE, APOC2
GO:0071827	plasma lipoprotein particle organization	2.85E-07	PLTP, LPL, CETP, APOE, APOC2
GO:0034375	high-density lipoprotein particle remodeling	3.27E-07	PLTP, CETP, APOE, APOC2
GO:0071825	protein-lipid complex subunit organization	3.27E-07	PLTP, LPL, CETP, APOE, APOC2
GO:0098754	detoxification	6.52E-07	MT1G, HP, HBG2, HBB, HBA2, APOE
GO:1905952	regulation of lipid localization	2.14E-06	ZC3H12A, PLTP, LPL, CETP, APOE, APOC2
GO:0042744	hydrogen peroxide catabolic process	2.56E-06	HP, HBG2, HBB, HBA2
GO:1905954	positive regulation of lipid localization	3.24E-06	ZC3H12A, PLTP, LPL, CETP, APOE
GO:0006898	receptor-mediated endocytosis	3.24E-06	CD163, VLDLR, HP, HBB, HBA2, APOE, APOC2
GO:0034371	chylomicron remodeling	3.96E-06	LPL, APOE, APOC2
GO:0055090	acylglycerol homeostasis	3.96E-06	LPL, CETP, APOE, APOC2
GO:0070328	triglyceride homeostasis	3.96E-06	LPL, CETP, APOE, APOC2
GO:0098869	cellular oxidant detoxification	3.96E-06	HP, HBG2, HBB, HBA2, APOE
GO:0034447	very-low-density lipoprotein particle clearance	5.18E-06	VLDLR, APOE, APOC2
GO:1990748	cellular detoxification	5.93E-06	HP, HBG2, HBB, HBA2, APOE
GO:0097237	cellular response to toxic substance	7.92E-06	HP, HBG2, HBB, HBA2, APOE
GO:0009636	response to toxic substance	1.01E-05	MT1G, HP, HBG2, HBB, HBA2, APOE
GO:0010876	lipid localization	1.38E-05	ZC3H12A, VLDLR, PLTP, LPL, CETP, APOE, APOC2
GO:0042743	hydrogen peroxide metabolic process	1.38E-05	HP, HBG2, HBB, HBA2
GO:0015671	oxygen transport	1.49E-05	HBG2, HBB, HBA2
GO:0033344	cholesterol efflux	2.04E-05	PLTP, CETP, APOE, APOC2
GO:0032374	regulation of cholesterol transport	2.82E-05	PLTP, CETP, APOE, APOC2
GO:0032371	regulation of sterol transport	2.87E-05	PLTP, CETP, APOE, APOC2
GO:0015669	gas transport	3.20E-05	HBG2, HBB, HBA2
GO:0043691	reverse cholesterol transport	3.60E-05	CETP, APOE, APOC2
GO:0015914	phospholipid transport	6.94E-05	PLTP, CETP, APOE, APOC2
GO:0033693	neurofilament bundle assembly	6.94E-05	NEFH, NEFM
GO:0032373	positive regulation of sterol transport	8.68E-05	PLTP, CETP, APOE
GO:0032376	positive regulation of cholesterol transport	8.68E-05	PLTP, CETP, APOE
GO:0042632	cholesterol homeostasis	9.43E-05	LPL, CETP, APOE, APOC2
GO:0055092	sterol homeostasis	9.54E-05	LPL, CETP, APOE, APOC2
GO:0030301	cholesterol transport	1.04E-04	PLTP, CETP, APOE, APOC2
GO:0006641	triglyceride metabolic process	1.22E-04	LPL, CETP, APOE, APOC2
GO:0015918	sterol transport	1.57E-04	PLTP, CETP, APOE, APOC2
GO:0015748	organophosphate ester transport	2.52E-04	PLTP, CETP, APOE, APOC2

ID	Description	qvalue	geneID
GO:0032368	regulation of lipid transport	2.52E-04	PLTP, CETP, APOE, APOC2
GO:0120009	intermembrane lipid transfer	2.52E-04	PLTP, CETP, APOE
GO:0072593	reactive oxygen species metabolic process	2.52E-04	ZC3H12A, HP, HBG2, HBB, HBA2
GO:0006638	neutral lipid metabolic process	2.52E-04	LPL, CETP, APOE, APOC2
GO:0006639	acylglycerol metabolic process	2.52E-04	LPL, CETP, APOE, APOC2
GO:0010874	regulation of cholesterol efflux	2.63E-04	PLTP, CETP, APOE
GO:0045110	intermediate filament bundle assembly	3.30E-04	NEFH, NEFM
GO:0008203	cholesterol metabolic process	3.55E-04	VLDLR, CYB5R3, CETP, APOE
GO:1902652	secondary alcohol metabolic process	4.34E-04	VLDLR, CYB5R3, CETP, APOE
GO:0055088	lipid homeostasis	4.36E-04	LPL, CETP, APOE, APOC2
GO:0016125	sterol metabolic process	4.92E-04	VLDLR, CYB5R3, CETP, APOE
GO:0034382	chylomicron remnant clearance	5.01E-04	APOE, APOC2
GO:0071830	triglyceride-rich lipoprotein particle clearance	5.01E-04	APOE, APOC2
GO:0034381	plasma lipoprotein particle clearance	7.10E-04	VLDLR, APOE, APOC2
GO:0034378	chylomicron assembly	7.10E-04	APOE, APOC2
GO:2001138	regulation of phospholipid transport	7.10E-04	CETP, APOE
GO:2001140	positive regulation of phospholipid transport	7.10E-04	CETP, APOE
GO:0032370	positive regulation of lipid transport	7.38E-04	PLTP, CETP, APOE
GO:0033700	phospholipid efflux	8.20E-04	APOE, APOC2
GO:0006869	lipid transport	8.20E-04	VLDLR, PLTP, CETP, APOE, APOC2
GO:0019915	lipid storage	1.08E-03	ZC3H12A, LPL, APOE
GO:0032802	low-density lipoprotein particle receptor catabolic process	1.08E-03	VLDLR, APOE
GO:0034374	low-density lipoprotein particle remodeling	1.40E-03	CETP, APOE
GO:0000302	response to reactive oxygen species	1.40E-03	HP, HBB, HBA2, APOE
GO:0034384	high-density lipoprotein particle clearance	1.53E-03	APOE, APOC2
GO:0006979	response to oxidative stress	1.53E-03	ZC3H12A, HP, HBB, HBA2, APOE
GO:0010875	positive regulation of cholesterol efflux	2.07E-03	PLTP, APOE
GO:0015850	organic hydroxy compound transport	2.18E-03	PLTP, CETP, APOE, APOC2
GO:0001523	retinoid metabolic process	2.24E-03	LPL, APOE, APOC2
GO:0016101	diterpenoid metabolic process	2.59E-03	LPL, APOE, APOC2
GO:0032799	low-density lipoprotein receptor particle metabolic process	2.60E-03	VLDLR, APOE

ID	Description	qvalue	geneID
GO:0051004	regulation of lipoprotein lipase activity	2.79E-03	LPL, APOC2
GO:0045109	intermediate filament organization	2.99E-03	NEFH, NEFM
GO:0006721	terpenoid metabolic process	3.14E-03	LPL, APOE, APOC2
GO:0010884	positive regulation of lipid storage	3.15E-03	ZC3H12A, LPL
GO:0044788	modulation by host of viral process	4.15E-03	ZC3H12A, APOE
GO:0008202	steroid metabolic process	4.32E-03	VLDLR, CYB5R3, CETP, APOE
GO:0034377	plasma lipoprotein particle assembly	4.32E-03	APOE, APOC2
GO:0006720	isoprenoid metabolic process	4.49E-03	LPL, APOE, APOC2
GO:0010743	regulation of macrophage derived foam cell differentiation	4.49E-03	LPL, CETP
GO:0042542	response to hydrogen peroxide	4.62E-03	HP, HBB, HBA2
GO:0055094	response to lipoprotein particle	4.95E-03	LPL, APOE
GO:0032801	receptor catabolic process	5.06E-03	VLDLR, APOE
GO:0065005	protein-lipid complex assembly	5.06E-03	APOE, APOC2
GO:1900016	negative regulation of cytokine production involved in inflammatory response	5.06E-03	ZC3H12A, PDCD4
GO:0071402	cellular response to lipoprotein particle stimulus	5.29E-03	LPL, APOE
GO:0010742	macrophage derived foam cell differentiation	5.70E-03	LPL, CETP
GO:0019433	triglyceride catabolic process	5.70E-03	LPL, APOC2
GO:0090077	foam cell differentiation	5.70E-03	LPL, CETP
GO:0006066	alcohol metabolic process	6.23E-03	VLDLR, CYB5R3, CETP, APOE
GO:0010189	vitamin E biosynthetic process	6.23E-03	PLTP
GO:0071831	intermediate-density lipoprotein particle clearance	6.23E-03	APOE
GO:0090731	cellular response to very-low-density lipoprotein particle stimulus	6.23E-03	APOE
GO:0150200	regulation of transport across blood-brain barrier	6.23E-03	APOE
GO:0150201	positive regulation of transport across blood-brain barrier	6.23E-03	APOE
GO:1903000	regulation of lipid transport across blood-brain barrier	6.23E-03	APOE
GO:1903002	positive regulation of lipid transport across blood-brain barrier	6.23E-03	APOE
GO:1905853	regulation of heparan sulfate binding	6.23E-03	APOE

ID	Description	qvalue	geneID
GO:1905855	positive regulation of heparan sulfate binding	6.23E-03	APOE
GO:1905858	regulation of heparan sulfate proteoglycan binding	6.23E-03	APOE
GO:1905860	positive regulation of heparan sulfate proteoglycan binding	6.23E-03	APOE
GO:1905890	regulation of cellular response to very-low-density lipoprotein particle stimulus	6.23E-03	APOE
GO:0015701	bicarbonate transport	6.24E-03	HBB, HBA2
GO:0090207	regulation of triglyceride metabolic process	6.24E-03	APOE, APOC2
GO:0006953	acute-phase response	7.38E-03	CD163, HP
GO:0046461	neutral lipid catabolic process	7.55E-03	LPL, APOC2
GO:0046464	acylglycerol catabolic process	7.55E-03	LPL, APOC2
GO:0050727	regulation of inflammatory response	7.72E-03	ZC3H12A, PDCD4, LPL, APOE
GO:0050728	negative regulation of inflammatory response	7.96E-03	ZC3H12A, PDCD4, APOE
GO:0045104	intermediate filament cytoskeleton organization	7.96E-03	NEFH, NEFM
GO:0046486	glycerolipid metabolic process	8.11E-03	LPL, CETP, APOE, APOC2
GO:0045103	intermediate filament-based process	8.13E-03	NEFH, NEFM
GO:2000377	regulation of reactive oxygen species metabolic process	8.20E-03	ZC3H12A, HP, HBB
GO:0010883	regulation of lipid storage	8.30E-03	ZC3H12A, LPL
GO:0071398	cellular response to fatty acid	9.87E-03	ZC3H12A, LPL
GO:0031638	zymogen activation	1.00E-02	C1RL, HP
GO:1903793	positive regulation of anion transport	1.00E-02	CETP, APOE
GO:0000294	nuclear-transcribed mRNA catabolic process, endonucleolytic cleavage-dependent decay	1.05E-02	ZC3H12A
GO:0010877	lipid transport involved in lipid storage	1.05E-02	APOE
GO:0010902	positive regulation of very-low-density lipoprotein particle remodeling	1.05E-02	APOC2
GO:2000296	negative regulation of hydrogen peroxide catabolic process	1.05E-02	HP
GO:0051851	modulation by host of symbiont process	1.09E-02	ZC3H12A, APOE
GO:0055081	anion homeostasis	1.09E-02	CETP, APOE
GO:2000378	negative regulation of reactive oxygen species metabolic process	1.22E-02	ZC3H12A, HP
GO:1900015	regulation of cytokine production involved in inflammatory response	1.25E-02	ZC3H12A, PDCD4

ID	Description	qvalue	geneID
GO:0045428	regulation of nitric oxide biosynthetic process	1.28E-02	ZC3H12A, HBB
GO:0002534	cytokine production involved in inflammatory response	1.33E-02	ZC3H12A, PDCD4
GO:0045600	positive regulation of fat cell differentiation	1.33E-02	ZC3H12A, LPL
GO:0046503	glycerolipid catabolic process	1.33E-02	LPL, APOC2
GO:0030185	nitric oxide transport	1.33E-02	HBB
GO:0032805	positive regulation of low-density lipoprotein particle receptor catabolic process	1.33E-02	APOE
GO:0048936	peripheral nervous system neuron axonogenesis	1.33E-02	NEFH
GO:0055095	lipoprotein particle mediated signaling	1.33E-02	LPL
GO:0055096	low-density lipoprotein particle mediated signaling	1.33E-02	LPL
GO:0060697	positive regulation of phospholipid catabolic process	1.33E-02	APOC2
GO:0060940	epithelial to mesenchymal transition involved in cardiac fibroblast development	1.33E-02	PDCD4
GO:0061771	response to caloric restriction	1.33E-02	APOE
GO:0099185	postsynaptic intermediate filament cytoskeleton organization	1.33E-02	NEFH
GO:1902994	regulation of phospholipid efflux	1.33E-02	APOE
GO:1902995	positive regulation of phospholipid efflux	1.33E-02	APOE
GO:1904761	negative regulation of myofibroblast differentiation	1.33E-02	PDCD4
GO:2000627	positive regulation of miRNA catabolic process	1.33E-02	ZC3H12A
GO:1901617	organic hydroxy compound biosynthetic process	1.33E-02	PLTP, CYB5R3, APOE
GO:0006695	cholesterol biosynthetic process	1.33E-02	CYB5R3, APOE
GO:1900006	positive regulation of dendrite development	1.33E-02	VLDLR, APOE
GO:1902653	secondary alcohol biosynthetic process	1.33E-02	CYB5R3, APOE
GO:0031348	negative regulation of defense response	1.45E-02	ZC3H12A, PDCD4, APOE
GO:0006809	nitric oxide biosynthetic process	1.45E-02	ZC3H12A, HBB
GO:0043407	negative regulation of MAP kinase activity	1.45E-02	PDCD4, APOE
GO:0016126	sterol biosynthetic process	1.51E-02	CYB5R3, APOE
GO:0046209	nitric oxide metabolic process	1.54E-02	ZC3H12A, HBB

ID	Description	qvalue	geneID
GO:0010916	negative regulation of very-low-density lipoprotein particle clearance	1.54E-02	APOC2
GO:0034436	glycoprotein transport	1.54E-02	VLDLR
GO:0042360	vitamin E metabolic process	1.54E-02	PLTP
GO:0060935	cardiac fibroblast cell differentiation	1.54E-02	PDCD4
GO:0060936	cardiac fibroblast cell development	1.54E-02	PDCD4
GO:0060938	epicardium-derived cardiac fibroblast cell differentiation	1.54E-02	PDCD4
GO:0060939	epicardium-derived cardiac fibroblast cell development	1.54E-02	PDCD4
GO:0097114	NMDA glutamate receptor clustering	1.54E-02	APOE
GO:1902952	positive regulation of dendritic spine maintenance	1.54E-02	APOE
GO:1904636	response to ionomycin	1.54E-02	ZC3H12A
GO:1904637	cellular response to ionomycin	1.54E-02	ZC3H12A
GO:1905908	positive regulation of amyloid fibril formation	1.54E-02	APOE
GO:2000295	regulation of hydrogen peroxide catabolic process	1.54E-02	HP
GO:2000625	regulation of miRNA catabolic process	1.54E-02	ZC3H12A
GO:2001057	reactive nitrogen species metabolic process	1.57E-02	ZC3H12A, HBB
GO:0046889	positive regulation of lipid biosynthetic process	1.59E-02	APOE, APOC2
GO:0070542	response to fatty acid	1.65E-02	ZC3H12A, LPL
GO:0002532	production of molecular mediator involved in inflammatory response	1.74E-02	ZC3H12A, PDCD4
GO:0051702	interaction with symbiont	1.74E-02	ZC3H12A, APOE
GO:0010660	regulation of muscle cell apoptotic process	1.76E-02	ZC3H12A, PDCD4
GO:1901264	carbohydrate derivative transport	1.76E-02	VLDLR, PLTP
GO:0010901	regulation of very-low-density lipoprotein particle remodeling	1.79E-02	APOC2
GO:0010915	regulation of very-low-density lipoprotein particle clearance	1.79E-02	APOC2
GO:1903936	cellular response to sodium arsenite	1.79E-02	ZC3H12A
GO:1904760	regulation of myofibroblast differentiation	1.79E-02	PDCD4
GO:1905907	negative regulation of amyloid fibril formation	1.79E-02	APOE
GO:0032651	regulation of interleukin-1 beta production	1.81E-02	ZC3H12A, LPL
GO:0010657	muscle cell apoptotic process	1.82E-02	ZC3H12A, PDCD4

ID	Description	qvalue	geneID
GO:0045833	negative regulation of lipid metabolic process	1.82E-02	APOE, APOC2
GO:0051817	modulation of process of other organism involved in symbiotic interaction	1.85E-02	ZC3H12A, APOE
GO:0060191	regulation of lipase activity	1.91E-02	LPL, APOC2
GO:0051235	maintenance of location	1.96E-02	ZC3H12A, LPL, APOE
GO:0044070	regulation of anion transport	1.99E-02	CETP, APOE
GO:0032803	regulation of low-density lipoprotein particle receptor catabolic process	1.99E-02	APOE
GO:0036446	myofibroblast differentiation	1.99E-02	PDCD4
GO:1903935	response to sodium arsenite	1.99E-02	ZC3H12A
GO:1990379	lipid transport across blood-brain barrier	1.99E-02	APOE
GO:2000630	positive regulation of miRNA metabolic process	1.99E-02	ZC3H12A
GO:2000646	positive regulation of receptor catabolic process	1.99E-02	APOE
GO:0032611	interleukin-1 beta production	1.99E-02	ZC3H12A, LPL
GO:2000379	positive regulation of reactive oxygen species metabolic process	1.99E-02	ZC3H12A, HBB
GO:1903426	regulation of reactive oxygen species biosynthetic process	2.01E-02	ZC3H12A, HBB
GO:0002526	acute inflammatory response	2.12E-02	CD163, HP
GO:0032652	regulation of interleukin-1 production	2.12E-02	ZC3H12A, LPL
GO:0007596	blood coagulation	2.12E-02	HBG2, HBB, APOE
GO:0016042	lipid catabolic process	2.12E-02	LPL, APOE, APOC2
GO:0035821	modulation of process of other organism	2.12E-02	ZC3H12A, APOE
GO:0007599	hemostasis	2.12E-02	HBG2, HBB, APOE
GO:0050817	coagulation	2.12E-02	HBG2, HBB, APOE
GO:0032489	regulation of Cdc42 protein signal transduction	2.12E-02	APOE
GO:0038026	reelin-mediated signaling pathway	2.12E-02	VLDLR
GO:0046836	glycolipid transport	2.12E-02	PLTP
GO:0060052	neurofilament cytoskeleton organization	2.12E-02	NEFH
GO:0060696	regulation of phospholipid catabolic process	2.12E-02	APOC2
GO:0071362	cellular response to ether	2.12E-02	ZC3H12A
GO:1900223	positive regulation of amyloid-beta clearance	2.12E-02	APOE
GO:1902075	cellular response to salt	2.12E-02	ZC3H12A
GO:1903003	positive regulation of protein deubiquitination	2.12E-02	ZC3H12A
GO:0051341	regulation of oxidoreductase activity	2.20E-02	HP, APOE

ID	Description	qvalue	geneID
GO:1901222	regulation of NIK/NF-kappaB signaling	2.26E-02	ZC3H12A, PDCD4
GO:0007613	memory	2.30E-02	VLDLR, APOE
GO:0032612	interleukin-1 production	2.30E-02	ZC3H12A, LPL
GO:0010727	negative regulation of hydrogen peroxide metabolic process	2.30E-02	HP
GO:0010886	positive regulation of cholesterol storage	2.30E-02	LPL
GO:0034196	acylglycerol transport	2.30E-02	CETP
GO:0034197	triglyceride transport	2.30E-02	CETP
GO:0044828	negative regulation by host of viral genome replication	2.30E-02	ZC3H12A
GO:0055118	negative regulation of cardiac muscle contraction	2.30E-02	ZC3H12A
GO:1905906	regulation of amyloid fibril formation	2.30E-02	APOE
GO:1903409	reactive oxygen species biosynthetic process	2.45E-02	ZC3H12A, HBB
GO:0010873	positive regulation of cholesterol esterification	2.45E-02	APOE
GO:0010890	positive regulation of sequestering of triglyceride	2.45E-02	LPL
GO:0010898	positive regulation of triglyceride catabolic process	2.45E-02	APOC2
GO:0045541	negative regulation of cholesterol biosynthetic process	2.45E-02	APOE
GO:0090206	negative regulation of cholesterol metabolic process	2.45E-02	APOE
GO:0097113	AMPA glutamate receptor clustering	2.45E-02	APOE
GO:0097688	glutamate receptor clustering	2.45E-02	APOE
GO:0106119	negative regulation of sterol biosynthetic process	2.45E-02	APOE
GO:1903365	regulation of fear response	2.45E-02	APOE
GO:2000343	positive regulation of chemokine (C-X-C motif) ligand 2 production	2.45E-02	LPL
GO:2000822	regulation of behavioral fear response	2.45E-02	APOE
GO:0006766	vitamin metabolic process	2.46E-02	PLTP, CYB5R3
GO:0031331	positive regulation of cellular catabolic process	2.54E-02	ZC3H12A, APOE, APOC2
GO:0010587	miRNA catabolic process	2.64E-02	ZC3H12A
GO:0042117	monocyte activation	2.64E-02	MT1G
GO:0045988	negative regulation of striated muscle contraction	2.64E-02	ZC3H12A
GO:2000320	negative regulation of T-helper 17 cell differentiation	2.64E-02	ZC3H12A
GO:2000644	regulation of receptor catabolic process	2.64E-02	APOE

ID	Description	qvalue	geneID
GO:0045598	regulation of fat cell differentiation	2.80E-02	ZC3H12A, LPL
GO:0006707	cholesterol catabolic process	2.80E-02	APOE
GO:0016127	sterol catabolic process	2.80E-02	APOE
GO:0019852	L-ascorbic acid metabolic process	2.80E-02	CYB5R3
GO:0051006	positive regulation of lipoprotein lipase activity	2.80E-02	APOC2
GO:0150104	transport across blood-brain barrier	2.80E-02	APOE
GO:1902513	regulation of organelle transport along microtubule	2.80E-02	NEFH
GO:2000317	negative regulation of T-helper 17 type immune response	2.80E-02	ZC3H12A
GO:2000628	regulation of miRNA metabolic process	2.80E-02	ZC3H12A
GO:0071901	negative regulation of protein serine/threonine kinase activity	2.91E-02	PDCD4, APOE
GO:0010232	vascular transport	2.97E-02	APOE
GO:0061365	positive regulation of triglyceride lipase activity	2.97E-02	APOC2
GO:0090085	regulation of protein deubiquitination	2.97E-02	ZC3H12A
GO:0090209	negative regulation of triglyceride metabolic process	2.97E-02	APOE
GO:1902950	regulation of dendritic spine maintenance	2.97E-02	APOE
GO:1905461	positive regulation of vascular associated smooth muscle cell apoptotic process	2.97E-02	PDCD4
GO:0045834	positive regulation of lipid metabolic process	3.01E-02	APOE, APOC2
GO:0032102	negative regulation of response to external stimulus	3.06E-02	ZC3H12A, PDCD4, APOE
GO:0050773	regulation of dendrite development	3.06E-02	VLDLR, APOE
GO:0030168	platelet activation	3.07E-02	HBB, APOE
GO:0050729	positive regulation of inflammatory response	3.07E-02	PDCD4, LPL
GO:0010745	negative regulation of macrophage derived foam cell differentiation	3.07E-02	CETP
GO:0035641	locomotory exploration behavior	3.07E-02	APOE
GO:0048934	peripheral nervous system neuron differentiation	3.07E-02	NEFH
GO:0048935	peripheral nervous system neuron development	3.07E-02	NEFH
GO:0055091	phospholipid homeostasis	3.07E-02	CETP
GO:0072578	neurotransmitter-gated ion channel clustering	3.07E-02	APOE

ID	Description	qvalue	geneID
GO:1900272	negative regulation of long-term synaptic potentiation	3.07E-02	APOE
GO:0032675	regulation of interleukin-6 production	3.09E-02	ZC3H12A, LPL
GO:0001933	negative regulation of protein phosphorylation	3.13E-02	ZC3H12A, PDCD4, APOE
GO:0046165	alcohol biosynthetic process	3.14E-02	CYB5R3, APOE
GO:0010872	regulation of cholesterol esterification	3.14E-02	APOE
GO:0010889	regulation of sequestering of triglyceride	3.14E-02	LPL
GO:0010896	regulation of triglyceride catabolic process	3.14E-02	APOC2
GO:0032488	Cdc42 protein signal transduction	3.14E-02	APOE
GO:0035627	ceramide transport	3.14E-02	PLTP
GO:0044793	negative regulation by host of viral process	3.14E-02	ZC3H12A
GO:0044794	positive regulation by host of viral process	3.14E-02	APOE
GO:0045472	response to ether	3.14E-02	ZC3H12A
GO:1902430	negative regulation of amyloid-beta formation	3.14E-02	APOE
GO:1903799	negative regulation of production of miRNAs involved in gene silencing by miRNA	3.14E-02	ZC3H12A
GO:1905064	negative regulation of vascular associated smooth muscle cell differentiation	3.14E-02	PDCD4
GO:0009896	positive regulation of catabolic process	3.17E-02	ZC3H12A, APOE, APOC2
GO:0006644	phospholipid metabolic process	3.18E-02	LPL, CETP, APOC2
GO:0032635	interleukin-6 production	3.21E-02	ZC3H12A, LPL
GO:0010273	detoxification of copper ion	3.21E-02	MT1G
GO:0020027	hemoglobin metabolic process	3.21E-02	AHSP
GO:0042159	lipoprotein catabolic process	3.21E-02	APOE
GO:0042362	fat-soluble vitamin biosynthetic process	3.21E-02	PLTP
GO:0051044	positive regulation of membrane protein ectodomain proteolysis	3.21E-02	APOE
GO:0072567	chemokine (C-X-C motif) ligand 2 production	3.21E-02	LPL
GO:0099188	postsynaptic cytoskeleton organization	3.21E-02	NEFH
GO:1900119	positive regulation of execution phase of apoptosis	3.21E-02	ZC3H12A
GO:1990169	stress response to copper ion	3.21E-02	MT1G
GO:2000341	regulation of chemokine (C-X-C motif) ligand 2 production	3.21E-02	LPL

ID	Description	qvalue	geneID
GO:0006633	fatty acid biosynthetic process	3.28E-02	LPL, APOC2
GO:0099173	postsynapse organization	3.28E-02	NEFH, APOE
GO:0031667	response to nutrient levels	3.35E-02	ZC3H12A, LPL, APOE
GO:0034380	high-density lipoprotein particle assembly	3.37E-02	APOE
GO:0043508	negative regulation of JUN kinase activity	3.37E-02	PDCD4
GO:0007409	axonogenesis	3.43E-02	VLDLR, NEFH, APOE
GO:0042326	negative regulation of phosphorylation	3.43E-02	ZC3H12A, PDCD4, APOE
GO:0030730	sequestering of triglyceride	3.43E-02	LPL
GO:0042953	lipoprotein transport	3.43E-02	APOC2
GO:0043117	positive regulation of vascular permeability	3.43E-02	APOE
GO:0045623	negative regulation of T-helper cell differentiation	3.43E-02	ZC3H12A
GO:0055089	fatty acid homeostasis	3.43E-02	APOE
GO:0061158	3'-UTR-mediated mRNA destabilization	3.43E-02	ZC3H12A
GO:0061687	detoxification of inorganic compound	3.43E-02	MT1G
GO:0070293	renal absorption	3.43E-02	HBB
GO:0097501	stress response to metal ion	3.43E-02	MT1G
GO:1902931	negative regulation of alcohol biosynthetic process	3.43E-02	APOE
GO:1902992	negative regulation of amyloid precursor protein catabolic process	3.43E-02	APOE
GO:0043312	neutrophil degranulation	3.43E-02	HP, HBB, CYB5R3
GO:0038061	NIK/NF-kappaB signaling	3.47E-02	ZC3H12A, PDCD4
GO:0002283	neutrophil activation involved in immune response	3.47E-02	HP, HBB, CYB5R3
GO:0043409	negative regulation of MAPK cascade	3.47E-02	PDCD4, APOE
GO:0010885	regulation of cholesterol storage	3.47E-02	LPL
GO:0034433	steroid esterification	3.47E-02	APOE
GO:0034434	sterol esterification	3.47E-02	APOE
GO:0034435	cholesterol esterification	3.47E-02	APOE
GO:0043031	negative regulation of macrophage activation	3.47E-02	ZC3H12A
GO:0044827	modulation by host of viral genome replication	3.47E-02	ZC3H12A
GO:0044872	lipoprotein localization	3.47E-02	APOC2
GO:1900221	regulation of amyloid-beta clearance	3.47E-02	APOE
GO:1902074	response to salt	3.47E-02	ZC3H12A
GO:1990000	amyloid fibril formation	3.47E-02	APOE
GO:0010544	negative regulation of platelet activation	3.59E-02	APOE
GO:0010744	positive regulation of macrophage derived foam cell differentiation	3.59E-02	LPL

ID	Description	qvalue	geneID
GO:0010985	negative regulation of lipoprotein particle clearance	3.59E-02	APOC2
GO:0034393	positive regulation of smooth muscle cell apoptotic process	3.59E-02	PDCD4
GO:1905288	vascular associated smooth muscle cell apoptotic process	3.59E-02	PDCD4
GO:1905459	regulation of vascular associated smooth muscle cell apoptotic process	3.59E-02	PDCD4
GO:2000319	regulation of T-helper 17 cell differentiation	3.59E-02	ZC3H12A
GO:0006694	steroid biosynthetic process	3.68E-02	CYB5R3, APOE
GO:0043112	receptor metabolic process	3.68E-02	VLDLR, APOE
GO:0010878	cholesterol storage	3.68E-02	LPL
GO:0045019	negative regulation of nitric oxide biosynthetic process	3.68E-02	ZC3H12A
GO:0060965	negative regulation of gene silencing by miRNA	3.68E-02	ZC3H12A
GO:0071243	cellular response to arsenic-containing substance	3.68E-02	ZC3H12A
GO:0097062	dendritic spine maintenance	3.68E-02	APOE
GO:1904406	negative regulation of nitric oxide metabolic process	3.68E-02	ZC3H12A
GO:0017038	protein import	3.72E-02	ZC3H12A, APOE
GO:0009110	vitamin biosynthetic process	3.82E-02	PLTP
GO:0010310	regulation of hydrogen peroxide metabolic process	3.82E-02	HP
GO:0043371	negative regulation of CD4-positive, alpha-beta T cell differentiation	3.82E-02	ZC3H12A
GO:0046890	regulation of lipid biosynthetic process	3.91E-02	APOE, APOC2
GO:0050866	negative regulation of cell activation	3.91E-02	ZC3H12A, APOE
GO:0071222	cellular response to lipopolysaccharide	3.94E-02	ZC3H12A, PDCD4
GO:0051000	positive regulation of nitric-oxide synthase activity	3.94E-02	APOE
GO:2000316	regulation of T-helper 17 type immune response	3.94E-02	ZC3H12A
GO:0030100	regulation of endocytosis	3.97E-02	APOE, APOC2
GO:0045723	positive regulation of fatty acid biosynthetic process	4.03E-02	APOC2
GO:0051043	regulation of membrane protein ectodomain proteolysis	4.03E-02	APOE

ID	Description	qvalue	geneID
GO:0051151	negative regulation of smooth muscle cell differentiation	4.03E-02	PDCD4
GO:0060149	negative regulation of posttranscriptional gene silencing	4.03E-02	ZC3H12A
GO:0060967	negative regulation of gene silencing by RNA	4.03E-02	ZC3H12A
GO:0071404	cellular response to low-density lipoprotein particle stimulus	4.03E-02	LPL
GO:1903798	regulation of production of miRNAs involved in gene silencing by miRNA	4.03E-02	ZC3H12A
GO:0050792	regulation of viral process	4.13E-02	ZC3H12A, APOE
GO:0071294	cellular response to zinc ion	4.17E-02	MT1G
GO:0090208	positive regulation of triglyceride metabolic process	4.17E-02	APOC2
GO:0031669	cellular response to nutrient levels	4.23E-02	ZC3H12A, LPL
GO:0071219	cellular response to molecule of bacterial origin	4.26E-02	ZC3H12A, PDCD4
GO:0016485	protein processing	4.28E-02	C1RL, HP
GO:0046639	negative regulation of alpha-beta T cell differentiation	4.28E-02	ZC3H12A
GO:0070920	regulation of production of small RNA involved in gene silencing by RNA	4.28E-02	ZC3H12A
GO:0035640	exploration behavior	4.44E-02	APOE
GO:0043903	regulation of symbiotic process	4.52E-02	ZC3H12A, APOE
GO:0002021	response to dietary excess	4.53E-02	APOE
GO:0006706	steroid catabolic process	4.53E-02	APOE
GO:0050996	positive regulation of lipid catabolic process	4.53E-02	APOC2
GO:0072539	T-helper 17 cell differentiation	4.53E-02	ZC3H12A
GO:2000353	positive regulation of endothelial cell apoptotic process	4.53E-02	PDCD4
GO:0045444	fat cell differentiation	4.59E-02	ZC3H12A, LPL
GO:0044242	cellular lipid catabolic process	4.60E-02	LPL, APOC2
GO:0032872	regulation of stress-activated MAPK cascade	4.60E-02	ZC3H12A, PDCD4
GO:0010894	negative regulation of steroid biosynthetic process	4.60E-02	APOE
GO:0045932	negative regulation of muscle contraction	4.60E-02	ZC3H12A
GO:0071280	cellular response to copper ion	4.60E-02	MT1G
GO:1905063	regulation of vascular associated smooth muscle cell differentiation	4.60E-02	PDCD4

ID	Description	qvalue	geneID
GO:0010594	regulation of endothelial cell migration	4.60E-02	ZC3H12A, APOE
GO:0034249	negative regulation of cellular amide metabolic process	4.60E-02	ZC3H12A, APOE
GO:0070302	regulation of stress-activated protein kinase signaling cascade	4.65E-02	ZC3H12A, PDCD4
GO:0002230	positive regulation of defense response to virus by host	4.68E-02	ZC3H12A
GO:0007263	nitric oxide mediated signal transduction	4.68E-02	APOE
GO:0045822	negative regulation of heart contraction	4.68E-02	ZC3H12A
GO:0048261	negative regulation of receptor-mediated endocytosis	4.68E-02	APOC2
GO:0006469	negative regulation of protein kinase activity	4.73E-02	PDCD4, APOE
GO:0071216	cellular response to biotic stimulus	4.73E-02	ZC3H12A, PDCD4
GO:0072330	monocarboxylic acid biosynthetic process	4.73E-02	LPL, APOC2
GO:0007271	synaptic transmission, cholinergic	4.73E-02	APOE
GO:0010586	miRNA metabolic process	4.73E-02	ZC3H12A
GO:0019934	cGMP-mediated signaling	4.73E-02	APOE
GO:0032372	negative regulation of sterol transport	4.73E-02	APOC2
GO:0032375	negative regulation of cholesterol transport	4.73E-02	APOC2
GO:0045939	negative regulation of steroid metabolic process	4.73E-02	APOE
GO:0016358	dendrite development	4.73E-02	VLDLR, APOE
GO:0032770	positive regulation of monooxygenase activity	4.81E-02	APOE
GO:0045940	positive regulation of steroid metabolic process	4.81E-02	APOE
GO:0072538	T-helper 17 type immune response	4.81E-02	ZC3H12A
GO:1900745	positive regulation of p38MAPK cascade	4.81E-02	ZC3H12A
GO:2000515	negative regulation of CD4-positive, alpha-beta T cell activation	4.81E-02	ZC3H12A
GO:0031668	cellular response to extracellular stimulus	4.87E-02	ZC3H12A, LPL
GO:0060317	cardiac epithelial to mesenchymal transition	4.93E-02	PDCD4
GO:0061157	mRNA destabilization	4.93E-02	ZC3H12A

Table S10: KEGG enriched pathways of R.M3

ID	Description	qvalue	geneID
hsa04979	Cholesterol metabolism	2.42E-07	PLTP, LPL, CETP, APOE, APOC2
hsa05143	African trypanosomiasis	1.19E-02	HBB, HBA2

ID	Description	qvalue	geneID
hsa05144	Malaria	1.44E-02	HBB, HBA2
hsa03320	PPAR signaling pathway	2.38E-02	PLTP, LPL

Table S11: DO enriched pathways of R.M3

ID	Description	qvalue	geneID
DOID:10241	thalassemia	6.19E-04	AHSP, HBG2, HBB, HBA2
DOID:3393	coronary artery disease	6.19E-04	CD163, PLTP, LPL, HP, CETP, APOE, APOC2
DOID:1387	hypolipoproteinemia	8.99E-03	LPL, APOE
DOID:10652	Alzheimer's disease	8.99E-03	PLTP, NEFM, LPL, HP, CETP, APOE
DOID:680	tauopathy	8.99E-03	PLTP, NEFM, LPL, HP, CETP, APOE
DOID:5844	myocardial infarction	8.99E-03	CD163, LPL, HP, CETP, APOE
DOID:589	congenital hemolytic anemia	1.26E-02	HBB, CYB5R3
DOID:4248	coronary stenosis	1.29E-02	CETP, APOE
DOID:1168	familial hyperlipidemia	1.29E-02	LPL, CETP, APOE
DOID:1936	atherosclerosis	1.29E-02	CD163, PLTP, LPL, CETP, APOE
DOID:2348	arteriosclerotic cardiovascular disease	1.29E-02	CD163, PLTP, LPL, CETP, APOE
DOID:2349	arteriosclerosis	1.34E-02	CD163, PLTP, LPL, CETP, APOE
DOID:3146	lipid metabolism disorder	1.34E-02	LPL, CETP, APOE
DOID:13809	familial combined hyperlipidemia	1.61E-02	LPL, APOE
DOID:12365	malaria	1.61E-02	HP, HBB, APOE
DOID:230	lateral sclerosis	1.75E-02	NEFH, NEFM, APOE
DOID:2355	anemia	1.75E-02	HBG2, HBB, HBA2, CYB5R3
DOID:10602	steatorrhea	1.75E-02	LPL
DOID:10783	methemoglobinemia	1.75E-02	CYB5R3
DOID:2789	parasitic protozoa infectious disease	2.20E-02	HP, HBB, APOE
DOID:3405	histiocytosis	2.25E-02	CD163, APOE
DOID:10923	sickle cell anemia	2.55E-02	HBB, HBA2
DOID:0050134	cutaneous mycosis	2.55E-02	APOE
DOID:14118	familial lipoprotein lipase deficiency	2.55E-02	LPL
DOID:3145	hyperlipoproteinemia type III	2.55E-02	LPL
DOID:8913	dermatophytosis	2.55E-02	APOE
DOID:1398	parasitic infectious disease	2.60E-02	HP, HBB, APOE
DOID:9970	obesity	2.70E-02	LPL, HP, CETP, APOE
DOID:1074	kidney failure	2.70E-02	CD163, HP, APOE
DOID:654	overnutrition	2.70E-02	LPL, HP, CETP, APOE
DOID:655	inherited metabolic disorder	2.70E-02	LPL, HBB, CETP, APOE
DOID:0050737	autosomal recessive disease	2.70E-02	AHSP, HBG2, HBB, HBA2
DOID:2860	hemoglobinopathy	2.70E-02	CYB5R3
DOID:8725	vascular dementia	2.70E-02	APOE
DOID:374	nutrition disease	2.70E-02	LPL, HP, CETP, APOE
DOID:1184	nephrotic syndrome	2.70E-02	CETP, APOE
DOID:9588	encephalitis	2.70E-02	CD163, APOE
DOID:3526	cerebral infarction	2.70E-02	LPL, APOE
DOID:2527	nephrosis	2.70E-02	CETP, APOE
DOID:1428	endocrine pancreas disease	2.70E-02	LPL, APOE
DOID:583	hemolytic anemia	2.70E-02	HBB, CYB5R3

ID	Description	qvalue	geneID
DOID:720	normocytic anemia	2.70E-02	HBB, CYB5R3
DOID:231	motor neuron disease	2.70E-02	NEFH, NEFM, APOE
DOID:3454	brain infarction	2.70E-02	LPL, APOE
DOID:10230	aortic atherosclerosis	2.70E-02	APOE
DOID:1099	alpha thalassemia	2.70E-02	HBA2
DOID:12978	Plasmodium vivax malaria	2.70E-02	HP
DOID:1386	abetalipoproteinemia	2.70E-02	APOE
DOID:8536	herpes zoster	2.70E-02	APOE
DOID:4247	coronary restenosis	3.31E-02	CETP
DOID:10871	age related macular degeneration	3.33E-02	VLDLR, APOE
DOID:2007	degeneration of macula and posterior pole	3.33E-02	VLDLR, APOE
DOID:4448	macular degeneration	3.44E-02	VLDLR, APOE
DOID:9352	type 2 diabetes mellitus	3.49E-02	PLTP, LPL, APOE
DOID:12549	hepatitis A	3.60E-02	APOE
DOID:12700	hyperprolactinemia	4.12E-02	LPL
DOID:12971	hereditary spherocytosis	4.55E-02	HBB
DOID:9279	hyperhomocysteinemia	4.55E-02	APOE
DOID:9452	fatty liver disease	4.81E-02	HBB, APOE
DOID:557	kidney disease	4.81E-02	CD163, HP, CETP, APOE
DOID:10459	common cold	4.81E-02	LPL
DOID:11476	osteoporosis	4.81E-02	HP, APOE
DOID:0080011	bone resorption disease	4.82E-02	HP, APOE
DOID:74	hematopoietic system disease	4.82E-02	HBG2, HBB, HBA2, CYB5R3
DOID:75	lymphatic system disease	4.82E-02	CD163, APOE
DOID:4449	macular retinal edema	4.82E-02	APOE
DOID:6929	retinal edema	4.82E-02	APOE
DOID:9993	hypoglycemia	4.82E-02	APOE
DOID:18	urinary system disease	4.82E-02	CD163, HP, CETP, APOE

S2.2.5 Significantly enriched pathways of R.M4

Table S12: GO-BP enriched pathways of R.M4

ID	Description	qvalue	geneID
GO:0002715	regulation of natural killer cell mediated immunity	1.74E-07	CD226, CD96, PVR
GO:0002228	natural killer cell mediated immunity	2.80E-07	CD226, CD96, PVR
GO:0002860	positive regulation of natural killer cell mediated cytotoxicity directed against tumor cell target	7.55E-07	CD226, PVR
GO:0002857	positive regulation of natural killer cell mediated immune response to tumor cell	7.55E-07	CD226, PVR
GO:0002420	natural killer cell mediated cytotoxicity directed against tumor cell target	7.55E-07	CD226, PVR
GO:0002858	regulation of natural killer cell mediated cytotoxicity directed against tumor cell target	7.55E-07	CD226, PVR

ID	Description	qvalue	geneID
GO:0002706	regulation of lymphocyte mediated immunity	7.55E-07	CD226, CD96, PVR
GO:0002370	natural killer cell cytokine production	7.55E-07	CD226, CD96
GO:0002423	natural killer cell mediated immune response to tumor cell	7.55E-07	CD226, PVR
GO:0002727	regulation of natural killer cell cytokine production	7.55E-07	CD226, CD96
GO:0002855	regulation of natural killer cell mediated immune response to tumor cell	7.55E-07	CD226, PVR
GO:0002836	positive regulation of response to tumor cell	1.35E-06	CD226, PVR
GO:0002839	positive regulation of immune response to tumor cell	1.35E-06	CD226, PVR
GO:0002703	regulation of leukocyte mediated immunity	1.35E-06	CD226, CD96, PVR
GO:0002834	regulation of response to tumor cell	1.73E-06	CD226, PVR
GO:0002837	regulation of immune response to tumor cell	1.73E-06	CD226, PVR
GO:0002418	immune response to tumor cell	2.58E-06	CD226, PVR
GO:0045088	regulation of innate immune response	3.24E-06	CD226, CD96, PVR
GO:0045954	positive regulation of natural killer cell mediated cytotoxicity	3.35E-06	CD226, PVR
GO:0002347	response to tumor cell	3.75E-06	CD226, PVR
GO:0002717	positive regulation of natural killer cell mediated immunity	4.46E-06	CD226, PVR
GO:0002449	lymphocyte mediated immunity	4.51E-06	CD226, CD96, PVR
GO:0002831	regulation of response to biotic stimulus	6.02E-06	CD226, CD96, PVR
GO:0042269	regulation of natural killer cell mediated cytotoxicity	7.87E-06	CD226, PVR
GO:0002697	regulation of immune effector process	8.41E-06	CD226, CD96, PVR
GO:0001912	positive regulation of leukocyte mediated cytotoxicity	1.17E-05	CD226, PVR
GO:0031343	positive regulation of cell killing	1.50E-05	CD226, PVR
GO:0042267	natural killer cell mediated cytotoxicity	1.50E-05	CD226, PVR
GO:0001910	regulation of leukocyte mediated cytotoxicity	2.20E-05	CD226, PVR
GO:0002718	regulation of cytokine production involved in immune response	2.61E-05	CD226, CD96
GO:0031341	regulation of cell killing	3.11E-05	CD226, PVR
GO:0002367	cytokine production involved in immune response	3.65E-05	CD226, CD96

ID	Description	qvalue	geneID
GO:0032649	regulation of interferon-gamma production	3.65E-05	CD226, CD96
GO:0002824	positive regulation of adaptive immune response based on somatic recombination of immune receptors built from immunoglobulin superfamily domains	3.65E-05	CD226, PVR
GO:0002708	positive regulation of lymphocyte mediated immunity	3.65E-05	CD226, PVR
GO:0001909	leukocyte mediated cytotoxicity	3.69E-05	CD226, PVR
GO:0002821	positive regulation of adaptive immune response	3.72E-05	CD226, PVR
GO:0032609	interferon-gamma production	3.96E-05	CD226, CD96
GO:0002705	positive regulation of leukocyte mediated immunity	5.56E-05	CD226, PVR
GO:0002700	regulation of production of molecular mediator of immune response	5.91E-05	CD226, CD96
GO:0002822	regulation of adaptive immune response based on somatic recombination of immune receptors built from immunoglobulin superfamily domains	6.51E-05	CD226, PVR
GO:0002819	regulation of adaptive immune response	7.66E-05	CD226, PVR
GO:0001906	cell killing	7.84E-05	CD226, PVR
GO:0002728	negative regulation of natural killer cell cytokine production	9.67E-05	CD96
GO:0060370	susceptibility to T cell mediated cytotoxicity	9.67E-05	PVR
GO:0045089	positive regulation of innate immune response	1.12E-04	CD226, PVR
GO:0002699	positive regulation of immune effector process	1.21E-04	CD226, PVR
GO:0042271	susceptibility to natural killer cell mediated cytotoxicity	1.36E-04	PVR
GO:0002833	positive regulation of response to biotic stimulus	1.46E-04	CD226, PVR
GO:0002440	production of molecular mediator of immune response	2.13E-04	CD226, CD96
GO:0060369	positive regulation of Fc receptor mediated stimulatory signaling pathway	2.99E-04	CD226

ID	Description	qvalue	geneID
GO:0002460	adaptive immune response based on somatic recombination of immune receptors built from immunoglobulin superfamily domains	2.99E-04	CD226, PVR
GO:0031349	positive regulation of defense response	3.17E-04	CD226, PVR
GO:0002729	positive regulation of natural killer cell cytokine production	3.22E-04	CD226
GO:0060368	regulation of Fc receptor mediated stimulatory signaling pathway	4.35E-04	CD226
GO:0050862	positive regulation of T cell receptor signaling pathway	5.82E-04	CD226
GO:0002716	negative regulation of natural killer cell mediated immunity	6.48E-04	CD96
GO:0033005	positive regulation of mast cell activation	8.24E-04	CD226
GO:0002719	negative regulation of cytokine production involved in immune response	8.84E-04	CD96
GO:0050857	positive regulation of antigen receptor-mediated signaling pathway	9.06E-04	CD226
GO:0001916	positive regulation of T cell mediated cytotoxicity	9.97E-04	PVR
GO:0001914	regulation of T cell mediated cytotoxicity	1.23E-03	PVR
GO:0002701	negative regulation of production of molecular mediator of immune response	1.24E-03	CD96
GO:0032689	negative regulation of interferon-gamma production	1.29E-03	CD96
GO:0002714	positive regulation of B cell mediated immunity	1.32E-03	CD226
GO:0002891	positive regulation of immunoglobulin mediated immune response	1.32E-03	CD226
GO:0002707	negative regulation of lymphocyte mediated immunity	1.36E-03	CD96
GO:0050856	regulation of T cell receptor signaling pathway	1.36E-03	CD226
GO:0001913	T cell mediated cytotoxicity	1.36E-03	PVR
GO:0033003	regulation of mast cell activation	1.36E-03	CD226
GO:0007157	heterophilic cell-cell adhesion via plasma membrane cell adhesion molecules	1.44E-03	PVR
GO:0002711	positive regulation of T cell mediated immunity	1.51E-03	PVR

ID	Description	qvalue	geneID
GO:0002704	negative regulation of leukocyte mediated immunity	1.55E-03	CD96
GO:0002720	positive regulation of cytokine production involved in immune response	1.60E-03	CD226
GO:0002712	regulation of B cell mediated immunity	1.60E-03	CD226
GO:0002889	regulation of immunoglobulin mediated immune response	1.60E-03	CD226
GO:0045576	mast cell activation	1.72E-03	CD226
GO:0045824	negative regulation of innate immune response	1.72E-03	CD96
GO:0032729	positive regulation of interferon-gamma production	1.78E-03	CD226
GO:0034332	adherens junction organization	1.82E-03	PVR
GO:0050854	regulation of antigen receptor-mediated signaling pathway	1.85E-03	CD226
GO:0002534	cytokine production involved in inflammatory response	1.85E-03	CD96
GO:0002709	regulation of T cell mediated immunity	1.91E-03	PVR
GO:0002532	production of molecular mediator involved in inflammatory response	2.40E-03	CD96
GO:0002832	negative regulation of response to biotic stimulus	2.50E-03	CD96
GO:0002702	positive regulation of production of molecular mediator of immune response	2.52E-03	CD226
GO:0002456	T cell mediated immunity	2.59E-03	PVR
GO:0002698	negative regulation of immune effector process	3.05E-03	CD96
GO:0046718	viral entry into host cell	3.25E-03	PVR
GO:0002431	Fc receptor mediated stimulatory signaling pathway	3.48E-03	CD226
GO:0044409	entry into host	3.49E-03	PVR
GO:0050777	negative regulation of immune response	3.73E-03	CD96
GO:0007156	homophilic cell adhesion via plasma membrane adhesion molecules	3.88E-03	PVR
GO:0052126	movement in host environment	3.88E-03	PVR
GO:0050852	T cell receptor signaling pathway	4.67E-03	CD226
GO:0045216	cell-cell junction organization	4.70E-03	PVR
GO:0051701	interaction with host	4.70E-03	PVR
GO:0008037	cell recognition	4.90E-03	CD226
GO:0016064	immunoglobulin mediated immune response	4.90E-03	CD226

ID	Description	qvalue	geneID
GO:0007160	cell-matrix adhesion	4.90E-03	CD96
GO:0019724	B cell mediated immunity	4.90E-03	CD226
GO:0031348	negative regulation of defense response	5.66E-03	CD96
GO:0098742	cell-cell adhesion via plasma-membrane adhesion molecules	5.77E-03	PVR
GO:0050851	antigen receptor-mediated signaling pathway	6.68E-03	CD226
GO:0032496	response to lipopolysaccharide	6.80E-03	CD96
GO:0019058	viral life cycle	6.87E-03	PVR
GO:0002237	response to molecule of bacterial origin	7.05E-03	CD96
GO:0031589	cell-substrate adhesion	7.05E-03	CD96
GO:0001818	negative regulation of cytokine production	7.05E-03	CD96
GO:0002696	positive regulation of leukocyte activation	7.86E-03	CD226
GO:0050867	positive regulation of cell activation	8.07E-03	CD226
GO:0032102	negative regulation of response to external stimulus	8.22E-03	CD96
GO:0001819	positive regulation of cytokine production	8.41E-03	CD226
GO:0002683	negative regulation of immune system process	8.62E-03	CD96
GO:0002429	immune response-activating cell surface receptor signaling pathway	8.80E-03	CD226
GO:0002757	immune response-activating signal transduction	8.80E-03	CD226

Table S13: KEGG enriched pathways of R.M4

ID	Description	qvalue	geneID
hsa04514	Cell adhesion molecules	<5.00E-02	CD226, PVR

Table S14: DO enriched pathways of R.M4

ID	Description	qvalue	geneID
DOID:4953	poliomyelitis	2.92E-03	PVR
DOID:8692	myeloid leukemia	2.92E-03	CD96, PVR
DOID:4226	endometrial stromal sarcoma	7.88E-03	CD226
DOID:5166	endometrial stromal tumor	8.97E-03	CD226
DOID:12132	Wegener's granulomatosis	8.97E-03	CD226
DOID:1380	endometrial cancer	2.84E-02	CD226
DOID:363	uterine cancer	2.84E-02	CD226
DOID:9119	acute myeloid leukemia	2.84E-02	CD96
DOID:12603	acute leukemia	2.84E-02	CD96

ID	Description	qvalue	geneID
DOID:865	vasculitis	2.84E-02	CD226
DOID:1575	rheumatic disease	3.31E-02	CD226
DOID:418	systemic scleroderma	3.31E-02	CD226
DOID:419	scleroderma	3.31E-02	CD226
DOID:854	collagen disease	3.31E-02	CD226

References

[1] S. P. Poulin, R. Dautoff, J. C. Morris, L. F. Barrett, B. C. Dickerson, A. D. N. Initiative, *et al.*, "Amygdala atrophy is prominent in early alzheimer's disease and relates to symptom severity," *Psychiatry Research: Neuroimaging*, vol. 194, no. 1, pp. 7–13, 2011.

[2] S. Kim, S. Swaminathan, M. Inlow, S. L. Risacher, K. Nho, L. Shen, T. M. Foroud, R. C. Petersen, P. S. Aisen, H. Soares, *et al.*, "Influence of genetic variation on plasma protein levels in older adults using a multi-analyte panel," *PloS one*, vol. 8, no. 7, p. e70269, 2013.

[3] A. J. Saykin, L. Shen, T. M. Foroud, S. G. Potkin, S. Swaminathan, S. Kim, S. L. Risacher, K. Nho, M. J. Huentelman, D. W. Craig, *et al.*, "Alzheimer's disease neuroimaging initiative biomarkers as quantitative phenotypes: Genetics core aims, progress, and plans," *Alzheimer's & Dementia*, vol. 6, no. 3, pp. 265–273, 2010.

[4] S. Purcell, B. Neale, K. Todd-Brown, L. Thomas, M. A. Ferreira, D. Bender, J. Maller, P. Sklar, P. I. De Bakker, M. J. Daly, *et al.*, "Plink: a tool set for whole-genome association and population-based linkage analyses," *The American journal of human genetics*, vol. 81, no. 3, pp. 559–575, 2007.

[5] V. K. Ramanan, S. L. Risacher, K. Nho, S. Kim, L. Shen, B. C. McDonald, K. K. Yoder, G. D. Hutchins, J. D. West, E. F. Tallman, *et al.*, "Gwas of longitudinal amyloid accumulation on 18f-florbetapir pet in alzheimer's disease implicates microglial activation gene il1rap," *Brain*, vol. 138, no. 10, pp. 3076–3088, 2015.

[6] L. Shen, S. Kim, S. L. Risacher, K. Nho, S. Swaminathan, J. D. West, T. Foroud, N. Pankratz, J. H. Moore, C. D. Sloan, *et al.*, "Whole genome association study of brain-wide imaging phenotypes for identifying quantitative trait loci in mci and ad: A study of the adni cohort," *Neuroimage*, vol. 53, no. 3, pp. 1051–1063, 2010.

[7] C. R. Jack Jr, M. A. Bernstein, N. C. Fox, P. Thompson, G. Alexander, D. Harvey, B. Borowski, P. J. Britson, J. L. Whitwell, C. Ward, *et al.*, "The alzheimer's disease neuroimaging initiative (adni): Mri methods," *Journal of Magnetic Resonance Imaging: An Official Journal of the International Society for Magnetic Resonance in Medicine*, vol. 27, no. 4, pp. 685–691, 2008.

[8] S. L. Risacher, A. J. Saykin, J. D. Wes, L. Shen, H. A. Firpi, and B. C. McDonald, "Baseline mri predictors of conversion from mci to probable ad in the adni cohort," *Current Alzheimer Research*, vol. 6, no. 4, pp. 347–361, 2009.

[9] N. Tzourio-Mazoyer, B. Landeau, D. Papathanassiou, F. Crivello, O. Etard, N. Delcroix, B. Mazoyer, and M. Joliot, "Automated anatomical labeling of activations in spm using a macroscopic anatomical parcellation of the mni mri single-subject brain," *Neuroimage*, vol. 15, no. 1, pp. 273–289, 2002.

[10] M. A. Reyna, M. D. Leiserson, and B. J. Raphael, "Hierarchical hotnet: identifying hierarchies of altered subnetworks," *Bioinformatics*, vol. 34, no. 17, pp. i972–i980, 2018.

[11] J. Lonsdale, J. Thomas, M. Salvatore, R. Phillips, E. Lo, S. Shad, R. Hasz, G. Walters, F. Garcia, N. Young, *et al.*, "The genotype-tissue expression (gtex) project," *Nature genetics*, vol. 45, no. 6, pp. 580–585, 2013.

[12] A. M. Kulminski, L. Shu, Y. Loika, L. He, A. Nazarian, K. Arbeev, S. Ukraintseva, A. Yashin, and I. Culminskaya, "Genetic and regulatory architecture of alzheimer's disease in the apoe region," *Alzheimer's & Dementia: Diagnosis, Assessment & Disease Monitoring*, vol. 12, no. 1, p. e12008, 2020.

[13] O. Belbin, J. L. Dunn, Y. Ling, L. Morgan, S. Chappell, H. Beaumont, D. Warden, D. A. Smith, N. Kalsheker, and K. Morgan, "Regulatory region single nucleotide polymorphisms of the apolipoprotein e gene and the rate of cognitive decline in alzheimer's disease," *Human molecular genetics*, vol. 16, no. 18, pp. 2199–2208, 2007.

[14] X. Tang, D. Holland, A. M. Dale, M. I. Miller, A. D. N. Initiative, *et al.*, "Apoe affects the volume and shape of the amygdala and the hippocampus in mild cognitive impairment and alzheimer's disease: age matters," *Journal of Alzheimer's Disease*, vol. 47, no. 3, pp. 645–660, 2015.

[15] L. Gao, Z. Cui, L. Shen, and H.-F. Ji, "Shared genetic etiology between type 2 diabetes and alzheimer's disease identified by bioinformatics analysis," *Journal of Alzheimer's Disease*, vol. 50, no. 1, pp. 13–17, 2016.

[16] A. I. Yashin, F. Fang, M. Kovtun, D. Wu, M. Duan, K. Arbeev, I. Akushevich, A. Kulminski, I. Culminskaya, I. Zhbannikov, *et al.*, "Hidden heterogeneity in alzheimer's disease: insights from genetic association studies and other analyses," *Experimental gerontology*, vol. 107, pp. 148–160, 2018.
